# Physicochemical Properties and Gut Microbiota-Regulating Activities of Macromolecules from Fresh and Dried Biyang *Flower Shiitake Mushrooms*: A Comparative Study Integrating 16S rRNA Sequencing and Untargeted Metabolomics

**DOI:** 10.3390/nu18142289

**Published:** 2026-07-13

**Authors:** Shunchao Zhang, Xiling Fan, Xinli Wei, Kai Li

**Affiliations:** 1 School of Management, Henan University of Chinese Medicine, Zhengzhou 450046, China; zyzyzsc@126.com; 2 College of Pharmacy, Henan University of Chinese Medicine, Zhengzhou 450046, China; fxl2020002061@163.com; 3 The First Affiliated Hospital of Henan University of Chinese Medicine, Zhengzhou 450046, China

**Keywords:** Biyang *flower shiitake mushroom* macromolecules, physicochemical properties, gut microbiota, untargeted metabolomics, structure-activity relationship

## Abstract

Background: Biyang *flower shiitake mushrooms* are valuable edible and medicinal fungi. However, the effects of macromolecules from fresh and dried *flower shiitake mushrooms* on the structural characteristics and their subsequent impact on gut health remain poorly understood. Methods: This study systematically investigated the physicochemical properties, microstructure, and thermal stability of macromolecules extracted from fresh (XG-PPC) and dried (GG-PPC) Biyang *flower shiitake mushrooms*. Furthermore, the differential regulatory effects of these supplements on the gut microbiota and fecal metabolome of mice were evaluated using 16S rRNA sequencing and untargeted metabolomics. Kendall’s rank correlation analysis was used to elucidate the intrinsic relationships among the physicochemical properties of macromolecules, key intestinal genera, and differential metabolites. Results: These structural differences conferred distinct biological effects on macromolecules. Protein and uronic acid contents act as key drivers governing the differentiation of gut microbiota and metabolic pathways, with opposing regulatory trends. Total carbohydrates, monosaccharide profiles, and molecular weight characteristics differentially regulate specific bacterial genera and multiple classes of metabolites, thereby establishing distinct gut microecological regulatory patterns between XG-PPC and GG-PPC. Conclusions: This study explores the structure-activity relationships of fresh and dried Biyang *shiitake mushroom* macromolecules, providing a theoretical foundation and empirical data to support the high-value utilization of Biyang *shiitake mushrooms* and the targeted development of corresponding functional foods.

## 1. Introduction

*Lentinula edodes* (Berk.) Pegler, classified within the genus *Lentinula* of the family *Omphalotaceae* [1], is an edible mushroom that derives nutrients for growth from lignin, cellulose, and hemicellulose in its substrate. It has significant edible and medicinal value [2]. *L. edodes* is rich in polysaccharides, proteins, amino acids, minerals, and vitamins [3,4,5]. Polysaccharides and proteins are the primary bioactive components of mushrooms and have attracted considerable research attention. Numerous studies have demonstrated that lentinan exhibits a broad spectrum of pharmacological properties, including anti-inflammatory, antioxidant, antitumor, and immunomodulatory effects [6,7,8,9]. Furthermore, it effectively regulates gut microbial composition and restores host microecological diversity [10,11], highlighting its considerable potential for application in pharmaceuticals, functional foods, and nutraceuticals.

*Flower shiitake mushrooms* are a high-quality species of the genus *Lentinus*, renowned both nationally and internationally for their large caps, thick flesh, rich aroma, and tenderness. They are characterized by significant edible and medicinal value and are often referred to as the “Queen of Plants” and the “Star of Fungi” [12,13]. *Floral mushrooms* from Biyang, China’s leading production region, are known for their unique color, aroma, texture, flavor, shape, pattern, and nutritional value. Consequently, they have earned the titles “Snow-white Flower Mushroom” and the “Emperor of Chinese Mushrooms” [14]. With an annual production of 60,000 tons of fresh *flower shiitake mushrooms*, accounting for 80% of China’s total output, Biyang exports mushrooms to more than 40 countries and regions. Having exceeded US $400 million in annual export revenue for five consecutive years, Biyang has established itself as a core global hub in the *flower shiitake mushrooms* supply chain. The county has developed a green industrial ecosystem characterized by the synergistic integration of fungal cultivation and agriculture, thereby creating a comprehensive cluster that integrates research and development, production, processing, and trade. Consequently, it has received multiple distinctions, including the title of Top Ten National Counties for Edible Fungi Production Bases [15]. However, research on Biyang *flower shiitake mushrooms* remains relatively scarce, and they have yet to be systematically and thoroughly investigated.

Currently, commercially available floral mushroom products are primarily categorized into fresh and dried types [16], with dried products accounting for up to 80% of the market [17]. Fresh *flower shiitake mushrooms* are characterized by a high moisture content and tender texture, exhibiting vigorous respiration and transpiration metabolism. These factors make them susceptible to browning, pileus opening, and decay, thereby limiting their storage and transportation. In contrast, modern drying techniques applied to *flower shiitake mushrooms* effectively reduce moisture content, extend shelf life, and enhance storage and transportation stability [18]. Notably, different processing methods not only alter the physical morphology of the materials but also induce significant changes in their chemical composition. Previous studies have demonstrated that various processing techniques substantially alter the chemical profiles of traditional Chinese medicines and foods, thereby affecting their biological activities [19,20,21]. Therefore, differences in the chemical compositions of fresh and dry *flower shiitake mushrooms* may lead to variations in their bioactivities.

As a central microbial ecosystem residing in the host gut, the intestinal microbiota performs essential physiological functions crucial to host health [22,23]. Beyond its fundamental roles in nutrient metabolism and energy acquisition, it actively participates in immune development and regulation, maintains intestinal barrier integrity [24], and, through metabolites such as short-chain fatty acids [25,26], facilitates cross-organ signaling along the gut–brain and gut–liver axes. These activities exert systemic effects on the metabolic, neural, and immune homeostasis of the host. Structurally diverse dietary macromolecules are essential prebiotics that modulate the gut microbiome by promoting beneficial taxa and inhibiting pathogenic strain growth. This precise modulation of microbial composition and function positions these microbes as promising therapeutic targets for enhancing host health. Numerous studies have demonstrated that naturally derived polysaccharides improve intestinal health by strengthening the intestinal barrier, reshaping gut microbiota composition, and increasing levels of beneficial metabolites [27,28,29], thereby positively influencing host well-being.

Based on the background described above, this study used fresh and dried *flower shiitake mushrooms* sourced from the Biyang area of Henan Province as raw materials. Their chemical composition, structural characteristics (analyzed by Fourier-transform infrared (FT-IR) and ultraviolet (UV) spectroscopy), micromorphology (examined via scanning electron microscopy (SEM) and atomic force microscopy (AFM)), and thermal stability of their macromolecules were systematically characterized. In addition, 16S rRNA high-throughput sequencing combined with untargeted metabolomics was used to investigate the differential regulatory effects of fresh and dried *flower shiitake mushrooms’* macromolecules on intestinal microflora homeostasis and associated metabolic pathways in mice. Subsequently, correlations among the physicochemical properties of macromolecules, key intestinal genera, and differential metabolites were evaluated. This study aimed to provide a theoretical foundation and experimental support for the high-value utilization of Biyang *flower shiitake mushrooms* and the development of functional foods.

## 2. Materials and Methods

### 2.1. Materials and Reagents

Biyang *flower shiitake mushrooms* employed in this study were purchased at the Mushroom Market in Biyang (Zhumadian, China) and were authenticated by Pro. Dong Chengming of Henan University of Chinese Medicine. Enhanced BCA Protein Assay Kit was purchased from Beyotime Biotechnology (Shanghai, China). SPF-grade male ICR mice were purchased from SBF Biotechnology Co., Ltd. (Suzhou, China), with the certificate number A202603100046. MagBeads FastDNA Kit for Soil was obtained from MP Biomedicals (Santa Ana, CA, USA). Quant-iT PicoGreen dsDNA Assay Kit was purchased from Thermo Fisher Scientific (Shanghai, China). 50% Sodium hydroxide (50% NaOH, GR grade) was procured from Alfa Aesar (Ward Hill, MA, USA). Sodium acetate (NaOAc, GR grade) was obtained from Thermo Fisher Scientific (Waltham, MA, USA). Methanol (MeOH), acetonitrile (ACN), and formic acid (all GR grade) were obtained from ANPEL Laboratory Technologies (Shanghai, China). All other reagents were of analytical grade (AR).

### 2.2. Animals and Treatments

Six- to eight-week-old male ICR mice (weight, 18–20 g; *n* = 15) were purchased from Sibeifu Biotechnology Co., Ltd. (Suzhou, China) (Certificate No. A20260310046). The animals were randomly assigned to three groups (*n* = 5 per group): Control, XG-PPC, and GG-PPC. Polysaccharide solutions were administered to the XG-PPC and GG-PPC groups via oral gavage at a dose of 200 mg/kg, and the Control group received an equivalent volume of distilled water. All treatments were administered once daily for five days. Fecal samples from all mice were collected on day 6 for subsequent analysis. Subsequently, all animals were euthanized by carbon dioxide inhalation. All animal experiments were conducted in accordance with the institutional guidelines for the care and use of laboratory animals and were approved by the relevant Institutional Animal Ethics Committee (Approval No. DWLL202203350).

### 2.3. Extraction of Macromolecules from Biyang Flower Shiitake Mushrooms

**Figure 1:**
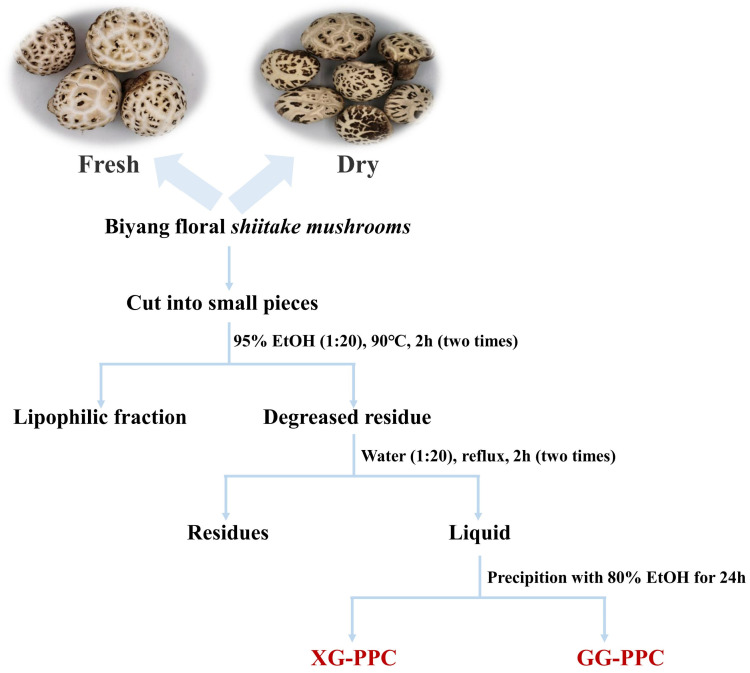
Flowchart of *macromolecular* extraction from Biyang *flower shiitake mushrooms*.

As shown in Figure 1, fresh and dried Biyang *flower shiitake mushrooms* (300 g each) were cut into small pieces and defatted by reflux extraction twice with 95% ethanol (1:20 *w*/*v*) at 90 °C for 2 h per extraction to remove lipophilic constituents. The resulting residue was extracted twice with distilled water at a ratio of 1:20 (*w*/*v*) under reflux for 2 h each time. The aqueous extracts were combined and concentrated under reduced pressure to an appropriate volume. Subsequently, four volumes of absolute ethanol were added, and the mixture was stored at 4 °C for 24 h to precipitate the macromolecules. After incubation, the supernatant was discarded, and the precipitate was collected by centrifugation at 4500 rpm for 10 min. The precipitated polysaccharides were washed twice with absolute ethanol, redissolved in distilled water, and dialyzed against running water for 48 h using a dialysis membrane with a molecular weight cut-off of 1000 Da (RuiDaHengHui Science & Technology Development Co., Ltd., Beijing, China). Finally, the dialyzed solution was lyophilized to obtain the macromolecular fractions, XG-PPC and GG-PPC.

### 2.4. Chemical Composition Analysis of XG-PPC and GG-PPC

Total carbohydrates were quantified using the phenol-sulfuric acid assay with glucose (Glc) as the calibration standard [30]. Protein concentrations were determined using the Enhanced BCA Protein Assay Kit, with bovine serum albumin as the reference [31]. Uronic acid levels were measured using the m-hydroxydiphenyl assay, with galacturonic acid as the standard [32].

### 2.5. Molecular Weight Analysis

XG-PPC and GG-PPC (10.0 mg each) were accurately weighed, dissolved, and diluted to 1.0 mL to obtain a 10.0 mg/mL solution. The solutions were filtered through a 0.22 μm microporous membrane and analyzed using an Agilent 1260 high-performance liquid chromatography system (Agilent, Santa Clara, CA, USA). The chromatographic columns used were Shodex Ohpak 806HQ (13 µm, 8.0 mm × 300 mm; Shodex, Tokyo, Japan) and Shodex Ohpak 804HQ (10 µm, 8.0 mm × 300 mm; Shodex, Japan), connected in series. The mobile phase consisted of 0.2 M NaCl and was delivered at a flow rate of 1.0 mL/min. Detection was performed using a refractive index detector (RID-G7162A) coupled with a multiangle light scattering detector (DAWN, Wyatt Technology Co., Goleta, CA, USA). This system was used to determine the weight-average molecular weight (Mw), number-average molecular weight (Mn), and polydispersity index (Mw/Mn) of polysaccharides. Data acquisition and calculations were performed using ASTRA 8 software.

### 2.6. Monosaccharide Composition Analysis

The monosaccharide composition of XG-PPC and GG-PPC were determined by using High performance anion electronic chromatography (HPAEC) with electrochemical detectors (ED) [33]. Briefly, 5 mg of each XG-PPC and GG-PPC was added to 3 M trifluoroacetic acid. The solution was then heated at 120 °C for three hours. The hydrolyzed products were thoroughly dissolved in 5 mL of double-distilled water using a vortex mixer. Subsequently, 25 or 50 μL of this solution was diluted with 975 or 950 μL of double-distilled water, respectively. One milliliter of the resulting solution was centrifuged at 12,000 rpm for 5 min use centrifuge (BioRidge, Shanghai, China). The supernatant was filtered through a 0.22 μm Millipore membrane. The samples were analyzed using HPAEC-PAD (ThermoFisher, Wilmington, DE, USA) equipped with a Dionex CarboPac PA20 column (150 × 3.0 mm). The mobile phase was delivered at a flow rate of 0.3 mL/min and consisted of the following eluents: A, water; B, 15 mM NaOH; and C, 15 mM NaOH with 100 mM NaOAc. The injection volume was 25 μL, and the column temperature was maintained at 30 °C. The elution gradient was programmed as follows: 0 min, A/B/C (98.8:1.2:0, *v*/*v*); 18 min, A/B/C (98.8:1.2:0, *v*/*v*); 20 min, A/B/C (50:50:0, *v*/*v*); 30 min, A/B/C (50:50:0, *v*/*v*); 30.1 min, A/B/C (0:0:100, *v*/*v*); 46 min, A/B/C (0:0:100, *v*/*v*); 46.1 min, A/B/C (0:100:0, *v*/*v*); 50 min, A/B/C (0:100:0, *v*/*v*); 50.1 min, A/B/C (98.8:1.2:0, *v*/*v*); and 80 min, A/B/C (98.8:1.2:0, *v*/*v*). Rhamnose (Rha), galacturonic acid, galactose (Gal), Glc, glucuronic acid (GlcA), arabinose (Ara), xylose (Xyl), fucose (Fuc), glucosamine hydrochloride, fructose, ribose, and galactosamine hydrochloride (all analytical grade) were purchased from Shanghai Yuanye Biotechnology Co., Ltd. Mannose (Man) (Shanghai, China) and Man were purchased from Sigma-Aldrich (St. Louis, MO, USA), respectively.

### 2.7. FT-IR and UV-Visible (Uv-Vis) Spectroscopy Analyses

Approximately 3.0 mg of each *flower shiitake mushroom* macromolecule was thoroughly mixed with an appropriate amount of chromatographic-grade potassium bromide powder in an agate mortar and pressed into a pellet using KBr as the background. Scanning analysis was performed using an INVENIO-S FT-IR spectrometer (Bruker, Mannheim, Germany) over the wavenumber range of 4000–450 cm^−1^, at a resolution of 4 cm^−1^ with 400 scans.

A spectrophotometer (UV2600, Shimadzu, Kyoto, Japan) was used to scan the UV-vis spectra of 1 mg/mL polysaccharide aqueous solutions in the wavelength range of 200–400 nm.

### 2.8. Thermal Analysis

The thermal stabilities of the macromolecules were evaluated using thermogravimetric analysis. Examining the thermal degradation profiles and mass-loss characteristics provides important information regarding their thermal stability, processing characteristics, and potential applications [34]. Approximately 2.0 mg of each *flower shiitake mushroom* macromolecule sample was accurately weighed into a sample pan, and its thermogravimetric properties were analyzed using a simultaneous thermal analyzer (STA 449 F5, NETZSCH, Selb, Germany). The measurement conditions included a temperature range from room temperature to 600 °C, a heating rate of 10 °C/min, and a scanning rate of 1 °C/min.

### 2.9. SEM and AFM Analyses

SEM and AFM are complementary tools for systematically characterizing macromolecule structures at both micro- and molecular scales. Consequently, these techniques are widely used to investigate the surface morphology and molecular conformations of macromolecule-based materials [35,36].

A small amount of the *flower shiitake mushroom* macromolecule powder was affixed to the conductive adhesive on the sample stage and sputter-coated with gold to ensure adequate electrical conductivity. SEM imaging was performed using a Quanta FEG 250 microscope (Thermo Fisher, Waltham, MA, USA) at an accelerating voltage of 15–30 kV, which was adjusted according to the conductivity of the sample. Energy-dispersive X-ray spectroscopy analysis was conducted using an Ultimax detector integrated with the SEM system.

The microstructure of the *flower shiitake mushroom* macromolecule was further analyzed using AFM. A macromolecule solution was prepared in ultrapure water at a concentration of 10 μg/mL. Subsequently, 10 μL of the solution was deposited onto a silicon wafer pretreated with anhydrous ethanol and air-dried at room temperature. After drying, the samples were imaged using a MultiMode 8-HR atomic force microscope (Bruker, Germany) operating in tapping mode with a Si_3_N_4_ probe. The resulting images were processed and analyzed using NanoScope Analysis 3.0 software (Bruker, Billerica, MA, USA).

### 2.10. Gut Microbiota Analysis

Total DNA was isolated from the fecal samples of the Control, XG-DT, and GG-DT groups. The purity and concentration of the extracted DNA were verified using agarose gel electrophoresis. The V3–V4 region of the 16S ribosomal RNA gene was then specifically amplified using the primers 338F (5′-barcode+ACTCCTACGGGAGGCAGCA-3′) and 806R (5′-GGACTACHVGGGTWTCTAAT-3′). Subsequent bioinformatics analyses were conducted using an online analysis platform provided by Sanshu Biotechnology (Nantong, Jiangsu, China) (https://www.sanshubio.com/index, accessed in 16 April 2026).

### 2.11. Untargeted Fecal Metabolomics Analysis

Approximately 50–100 mg of fecal sample was extracted with 1.6 mL of pre-cooled methanol-acetonitrile-water (2:2:1, *v*/*v*/*v*) by vortexing and sonication. Proteins were precipitated at −20 °C for 1 h, followed by centrifugation at 4 °C and 12,000 rpm for 10 min. The resulting supernatant was dried, lyophilized, and reconstituted in 200 μL of methanol. After the final centrifugation, the supernatant was collected for analysis. Samples were analyzed by UPLC-Orbitrap-MS using a Waters HSS T3 (Waters, Milford, MA, USA) column (100 × 2.1 mm, 1.8 μm) maintained at 40 °C, with a flow rate of 0.3 mL/min and an injection volume of 2 μL. The mobile phase consisted of water with 0.1% acetic acid (A) and acetonitrile with 0.1% acetic acid (B), using the following gradient: 0–1 min, 100% A; 4 min, 60% A/40% B; 6.5 min, 5% A/95% B; 6.6–8.0 min, 100% A. HRMS data were acquired on a Q Exactive HFX in Full-ms-ddMS2 mode with a HESI source using the following parameters: spray voltage −2.8/+3.0 kV; sheath/auxiliary/sweep gas flow rates set to 40/10/0 arbitrary units; capillary temperature 320 °C; auxiliary heater temperature 350 °C. MS data were acquired using a Q Exactive and processed using Progenesis QI software (3.0.7927, Waters, USA). Subsequent bioinformatics analysis was conducted using an online analysis platform provided by Sanshu Biotechnology (https://www.sanshubio.com/index, accessed in 15 April 2026).

### 2.12. Correlation Analysis of Physicochemical Properties, Gut Microbiota, and Differential Metabolites of Floral Mushroom Polysaccharides

Kendall’s rank correlation analysis was performed to generate a correlation heatmap based on physicochemical properties, key gut bacterial genera, and the abundance of significant differential metabolites. This analysis aimed to clarify the potential relationships between macromolecule structural characteristics, gut microbiota composition, and metabolites. The nonparametric Kruskal–Wallis H test was applied to data violating assumptions of normality or homogeneity of variance, followed by Dunn’s post hoc test for intergroup pairwise comparisons.

### 2.13. Statistical Analysis

Statistical analyses were performed using GraphPad Prism (version 8.0, GraphPad Software, LLC, San Diego, CA, USA). All data are presented as mean ± standard deviation. For three-group comparisons, one-way analysis of variance (ANOVA) followed by Tukey’s post hoc test was used for data with normal distribution and homogeneity of variance. Differences where *p* < 0.05 were considered statistically significant, ns represents non-significant difference.

## 3. Result

### 3.1. Extraction Yields and Physicochemical Properties of XG-PPC and GG-PPC

As shown in Table 1, the macromolecule extraction yield of XG-PPC was 0.81%, whereas that of GG-PPC was 2.13%. Given the substantial difference in moisture content between fresh and dried Biyang floral *shiitake mushrooms* specified in GB/T 22746-2008 [37], these yield values are excluded from subsequent comparative analyses. The total carbohydrate content, determined using the phenol-sulfuric acid method, was 31.56% for XG-PPC and 34.80% for GG-PPC. The corresponding protein contents were 37.99% and 35.46%, respectively, whereas the uronic acid contents, measured using the m-hydroxydiphenyl method, were 4.27% and 6.90%, respectively.

**Figure 2:**
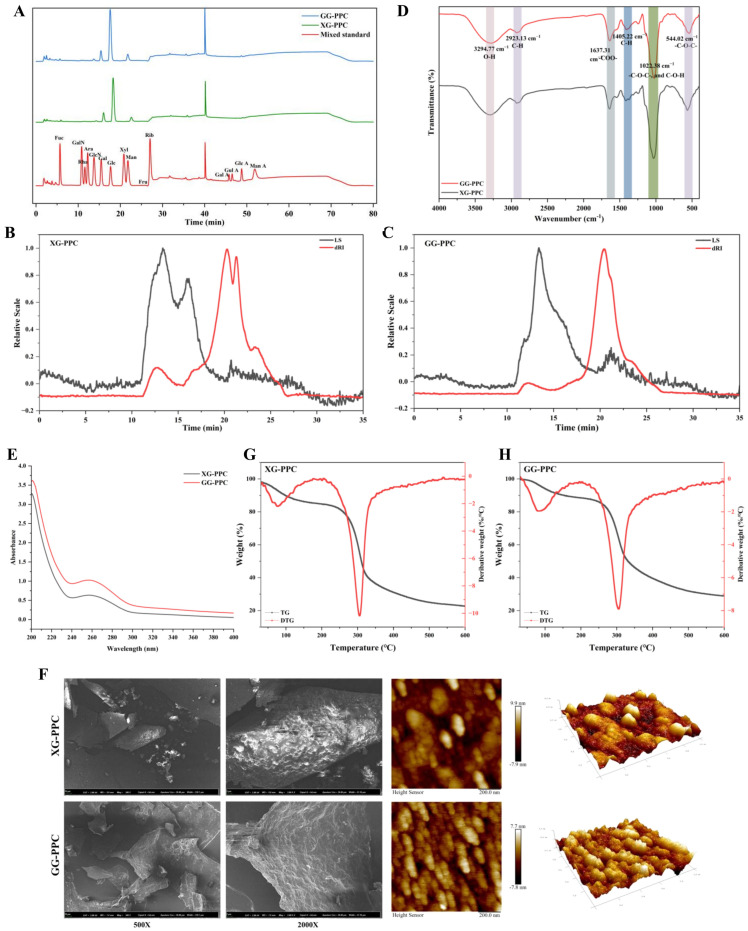
(**A**) Monosaccharide composition, (**B**,**C**) Chromatograms of the molar mass distribution of XG-PPC and GG-PPC, (**D**) FT-IR spectra, (**E**) UV–vis spectra, (**F**) SEM features, and 2D and 3D AFM images of XG-PPC and GG-PPC, (**G**,**H**) Thermal stability properties of XG-PPC and GG-PPC.

Monosaccharide composition analysis revealed that XG-PPC consists of 10 monosaccharides: Glc, Gal, Man, Fuc, Rha, Ara, Xyl, Glu, Gal N, and Glc A. Among these, Glc was identified as the predominant monosaccharide, with a molar ratio of 0.805 and a content of 526.913 μg/mg. Gal and Man were minor components, exhibiting molar ratios of 0.105 and 0.049, with contents of 68.625 μg/mg and 32.252 μg/mg, respectively. Rib, Gal A, Gul A, and Man A were not detected (Figure 2A, Table 1). GG-PPC comprises 12 monosaccharides: Glc, Gal, Man, Rib, Glc N, Fuc, Rha, Ara, Xyl, Gal N, Gal A, and GlcA. Among these, Glc was the predominant component, with a molar ratio of 0.774 and a content of 314.345 μg/mg. Gal and Man were secondary components, with molar ratios of 0.092 and 0.060 and corresponding contents of 37.538 μg/mg and 24.327 μg/mg, respectively (Figure 2A, Table 1). The molar ratios of the remaining monosaccharides were all below 0.020, accompanied by low contents, indicating that GG-PPC is a heteropolysaccharide predominantly composed of Glc. Both polysaccharides are heteropolysaccharides predominantly composed of Glc and display comparable profiles of monosaccharide composition and molar ratios, suggesting structural homology at the level of primary composition. However, distinct variations were observed in the composition of the minor constituents and acidic sugars between the two samples. Specifically, Rib and Gal A were detected in GG-PPC but not in XG-PPC (Figure 2A). Moreover, XG-PPC exhibited significantly higher Glc and lower Glc A levels than GG-DT (Table 1). These discrepancies suggest that XG-PPC and GG-PPC vary in acidic sugar residue retention, which may underlie their distinct biological activities.

### 3.2. Molecular Weight Analysis

The average molecular weight and molecular weight distribution of the XG-PPC and GG-PPC were determined using HPSEC-MALLS-RI. The results revealed that (Figure 2B,C) Mn, Mw, and Mw/Mn of XG-PPC were 2.021 × 10^5^ g/mol, 3.186 × 10^5^ g/mol, and 1.576, respectively, indicating a relatively uniform molecular weight distribution. (Table 1). For GG-PPC, Mn was 4.097 × 10^4^ g/mol, Mw was 3.891 × 10^5^ g/mol, and the polydispersity index (Mw/Mn) was 9.496, indicating a broad molecular weight distribution with a higher proportion of high-molecular-weight components (Table 1). Overall, the two macromolecules exhibited similar Mw values, whereas GG-PPC demonstrated a significantly higher polydispersity than XG-PPC. These findings suggest notable differences in their chain length distributions and degrees of polymerization, which may influence their physicochemical properties and biological activities, thereby providing a critical foundation for subsequent structure–activity relationship studies.

### 3.3. FT-IR and UV-Vis Analyses

FT-IR spectroscopy was used to characterize the functional groups (Figure 2D) [38]. The intense and broad peak at approximately 3300 cm^−1^ was attributed to O–H stretching vibrations, whereas the strong signal near 2920 cm^−1^ corresponded to C–H stretching vibrations. The absorption peak at approximately 1630 cm^−1^ was assigned to the asymmetric and symmetric stretching vibrations of the –COO-group, suggesting the presence of GlcA in XG-PPC and GG-PPC. The peak observed near 1405 cm^−1^ corresponded to C–H bending vibrations, whereas the strong absorption bands between 1100 and 1000 cm^−1^ were characteristic of C–O–C glycosidic bonds and C–O–H side group vibrations of the pyranose ring, respectively. The peak at 544.02 cm^−1^ was attributed to the low-frequency bending vibration of C–O–C glycosidic bonds, which is a key feature of the pyranose ring skeleton in polysaccharide structures. Overall, the FT-IR spectra of XG-DT and GG-DT showed no significant differences, confirming the high similarity in their functional group composition and backbone structure and indicating that drying had a negligible impact on their characteristic functional groups.

As shown in Figure 2E, both XG-PPC and GG-PPC exhibited strong absorption in the 200–220 nm range, which is a typical UV characteristic of sugar residues in polysaccharide structures, confirming that both samples have a polysaccharide backbone. In addition, both macromolecules displayed weak absorption peaks at approximately 260–280 nm, which are commonly attributed to the presence of proteins and/or nucleic acids. Together with the previous protein content determination results, this further confirms that both macromolecules contain bound proteins.

### 3.4. Surface Morphological Properties

We characterized and compared the macroscopic morphology and surface microstructures of XG-PPC and GG-PPC using SEM (Figure 2F). At a magnification of 500×, both macromolecules exhibited an irregular distribution of sheet-like and block-like structures; however, significant differences in their morphological features were observed. The GG-PPC fragments exhibited well-defined margins, no apparent adhesion or agglomeration, and a relatively uniform particle size distribution. In contrast, the XG-PPC fragments exhibited more fragmented and irregular edges, with numerous fine granular aggregates attached to the surface or scattered around the sample, indicating pronounced particle adhesion and agglomeration. At a magnification of 2000×, the differences in the surface microstructures of the two macromolecules became more pronounced. The GG-PPC surface exhibited a fine, uniformly wrinkled texture, lacking distinct pores or cracks, and a dense, well-integrated structure. In contrast, the XG-PPC surface was rough and uneven, characterized by numerous protrusions, pores, and irregular flocculent deposits, indicating a loose structure and poor structural uniformity.

AFM was used to characterize the micromorphology and surface roughness of the XG-PPC and GG-PPC samples (Figure 2F). The two-dimensional height images and three-dimensional topographical maps obtained from AFM revealed that GG-PPC macromolecule particles exhibited discrete, uniformly distributed ellipsoidal structures with well-defined interparticle boundaries and no noticeable adhesion or bridging. In contrast, XG-PPC macromolecule particles displayed significant interparticle adhesion and aggregation, characterized by less distinct particle boundaries and the fusion of multiple particles into irregular aggregates, resulting in lower morphological uniformity than that of GG-PPC particles.

In conclusion, significant differences were observed in the microscopic morphology and aggregation behaviour of the two macromolecules. Drying treatment effectively reduced particle aggregation and adhesion in the *flower shiitake mushroom* macromolecule, resulting in improved particle dispersion and structural homogeneity. These differences in microstructural characteristics may affect solubility, microbial accessibility, and interactions with gut microbiota, potentially contributing to the observed differences in prebiotic activity and gut microbiota modulation. Collectively, these results provide a structural basis for understanding the distinct biological activities of XG-PPC and GG-PPC.

### 3.5. Thermal Analysis

Thermogravimetric analysis was used to evaluate and characterize the thermal stabilities of XG-PPC and GG-PPC. As shown in Figure 2G,H, both samples exhibited typical three-stage thermal decomposition processes but showed distinct differences in their thermal stability profiles. The first stage corresponds to dehydration, which involves the removal of bound water located on the surface and entrapped within macromolecules. The mass loss of XG-PPC between 30 and 150 °C was 12.13%, which was attributed to its loose structure and hygroscopic morphology (Figure 2G). In contrast, GG-PPC exhibited a mass loss of 10.54% between 30 and 180 °C (Figure 2H), which was significantly lower than that of XG-PPC, suggesting that the drying treatment effectively reduced both the moisture adsorption capacity and bound water content in the macromolecules. The second stage represents the primary thermal decomposition of the macromolecule backbone. XG-PPC underwent rapid degradation between 150 and 320 °C, with a mass loss of 42.92%, whereas GG-PPC decomposed primarily between 180 and 350 °C, with a mass loss of 42.87% (Figure 2G). Notably, the decomposition onset temperature of GG-PPC was significantly higher than that of XG-PPC, and its pyrolysis process was more gradual than that of XG-PPC. The third stage involved the slow oxidation and carbonization of pyrolysis residues. The final residual mass of XG-PPC between 320 and 600 °C was 22.66% (Figure 2G), whereas GG-PPC retained 28.93% between 350 and 600 °C, which was slightly higher than that of XG-PPC (Figure 2H). This suggests that the pyrolysis products of GG-PPC exhibited greater thermal stability. In conclusion, GG-PPC exhibited a significantly broader thermal stability range than XG-PPC, characterized by a higher onset decomposition temperature and more gradual thermal degradation. These findings suggest that the drying process effectively enhances the thermal stability of Biyang *flower shiitake mushroom* macromolecules by modulating molecular aggregation and strengthening intermolecular interactions, without compromising the primary macromolecule structure. These results provide important thermal evidence supporting the suitability of these materials for subsequent processing and their structural stability in the intestinal environment.

### 3.6. Regulatory Effects of XG-PPC and GG-PPC on the Structure and Function of the Mouse Gut Microbiota

To systematically investigate the modulatory effects of XG-PPC and dried GG-PPC on the gut microbiome of mice, we conducted a comprehensive analysis using 16S rRNA gene high-throughput sequencing. Multiple aspects were examined, including alpha and beta diversity, community composition at the phylum and genus levels, Linear Discriminant Analysis Effect Size (LEfSe) analysis to identify differentially abundant taxa, and predicted functional profiles based on the Kyoto Encyclopedia of Genes and Genomes (KEGG) database.

**Figure 3:**
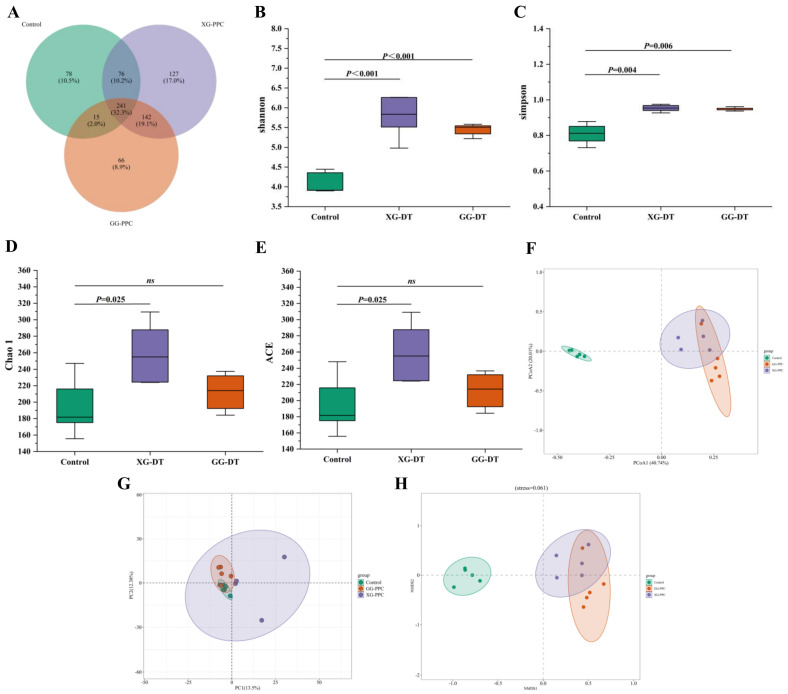
XG-PPC and GG-PPC reshaped the gut microbiota of mice. (**A**) Venn diagram of operational taxonomic units (OTUs) of gut microbiota in mice from the control group, XG-PPC group, and GG-PPC group, showing the number and proportion of shared and unique OTUs. (**B**–**E**) Analysis of gut microbiota α-diversity indices: (**B**) Shannon Index, (**C**) Simpson Index, (**D**) Chao1 Index, (**E**) ACE Index. (**F**–**H**) β-diversity analysis of gut microbiota, including (**F**) Principal Coordinates Analysis (PCoA, based on the Bray–Curtis distance algorithm), (**G**) Principal Component Analysis (PCA), and (**H**) Non-metric Multidimensional Scaling (NMDS), illustrating the overall separation trend of microbial community structures among different groups. ns denotes no significant difference.

Analysis of α-diversity indices revealed that (Figure 3B,C) treatment with either XG-PPC or GG-PPC significantly increased the Shannon (XG-PPC: *p* = 0.004; GG-PPC: *p* = 0.006) and Simpson (XG-PPC: *p* < 0.001; GG-PPC: *p* < 0.001) indices of murine gut microbiota compared with those in the Control group. This indicates that both Biyang *flower shiitake mushroom* macromolecules enhanced microbial diversity and community evenness, thereby supporting a more balanced intestinal microbial ecosystem. Further analysis of richness-related indices showed that (Figure 3D,E) the Chao1 (*p* = 0.025) and ACE (*p* = 0.025) indices were significantly higher in the XG-PPC group than in the Control group, whereas no significant differences were observed between the GG-PPC and Control groups. This finding suggests that XG-PPC significantly promoted microbial richness, whereas GG-PPC had no effect on microbial richness. Collectively, these findings demonstrate that Biyang *flower shiitake mushroom* macromolecules positively modulate the gut microbial community structure, with XG-PPC and GG-PPC exhibiting distinct effects on microbial richness and diversity.

This study used three methods, Principal Coordinates Analysis (PCoA), principal component analysis (PCA), and Non-metric Multidimensional Scaling (NMDS), to comprehensively assess β-diversity patterns and characterize the differences in the gut microbial community structure among the treatment groups. As shown in Figure 3F, PCoA based on the Bray–Curtis distance matrix revealed that the first two PCoAs collectively accounted for 60.75% of the total variation in gut microbial community composition, with PCoA1 and PCoA2 explaining 40.74% and 20.01% of the variation, respectively. The ordination plot demonstrated distinct clustering of samples among the three groups, particularly along the PCoA1 axis. Specifically, samples from the Control group were predominantly distributed on the negative side of PCoA1, whereas samples from the XG-PPC and GG-PPC groups were located on the positive side, forming distinct treatment-associated clusters. Notably, the GG-PPC group exhibited greater intra-group clustering than the XG-PPC group, suggesting a more homogeneous microbial community composition within this treatment group. These results suggest that dietary supplementation with both macromolecules significantly altered the overall structure of the murine gut microbiota, with GG-PPC exerting a more pronounced effect on reshaping microbial community composition. Similar patterns were observed in the PCA, in which the first two PCs explained 13.50% and 12.38% of the total variation. The Control and GG-PPC groups exhibited clear separation in the ordination space, whereas samples from the XG-PPC group were more widely dispersed, with some overlapping the Control group. This suggests greater intragroup heterogeneity in the gut microbiota structure of the XG-PPC group, whereas the GG-PPC group demonstrated a higher structural homogeneity. The NMDS analysis yielded a stress value of 0.061, which was below the 0.1 threshold as shown in Figure 3H, indicating the high reliability of the ordination results. The samples from the Control and two polysaccharide intervention groups were distinctly separated along the NMDS1 axis: the control group clustered on the left side, whereas the XG-PPC and GG-PPC groups were clustered on the right. In addition, the samples in the GG-PPC group showed greater aggregation, further supporting the findings of the PCoA and PCA analyses. These results confirm that both XG-PPC and GG-PPC significantly altered the overall gut microbiota structure in mice, with GG-PPC exerting a more stable and pronounced effect on remodeling the microbial community. The three β-diversity analyses corroborated each other, collectively demonstrating that compared with the control group, intervention with both fresh and dried Biyang *flower shiitake mushroom* macromolecules significantly modified murine gut microbiota structure. Furthermore, distinct differences were observed in the regulatory effects of the two macromolecules, with GG-PPC having a more substantial impact on reshaping the gut microbial community structure.

**Figure 4:**
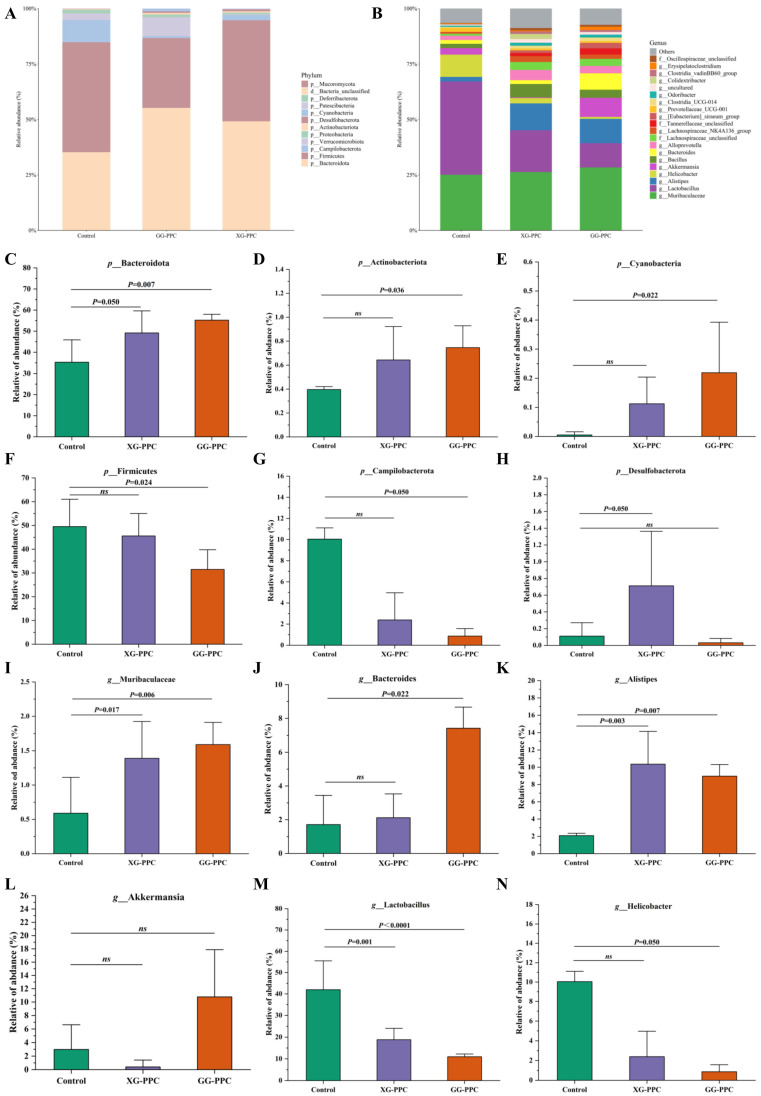
(**A**,**B**) Relative abundance distribution of gut microbiota at the phylum level and genus level. (**A**) Phylum level, (**B**) Genus level. (**C**–**H**) Quantitative analysis of the relative abundance of key bacterial phyla. (**C**) *p_Bacteroidota*, (**D**) *p_Actinobacteriota*, (**E**) *p_Cyanobacteria*, (**F**) *p_Firmicutes*, (**G**) *p_Campilobacterota*, and (**H**) *p_Desulfobacterota*. (**I**–**N**) Quantitative analysis of the relative abundance of key bacterial genera. (**I**) *g_Muribaculaceae*, (**J**) *g_Bacteroides*, (**K**) *g_Alistipes*, (**L**) *g_Akkermansia*, (**M**) *g_Lactobacillus*, (**N**) *g_Helicobacter*. ns denotes no significant difference.

At the phylum level (Figure 4A), XG-PPC and GG-PPC induced distinct alterations in the relative abundance of the mouse gut microbiota. Compared with the Control group, GG-PPC had more pronounced regulatory effects on the microbial community, whereas XG-PPC caused only moderate changes. This suggests that the regulatory effects of shiitake polysaccharides on the gut microbiota depend on processing-induced structural variations. GG-PPC broadly regulated intestinal homeostasis by significantly increasing the relative abundance of beneficial phyla, including *p_Bacteroidota* (*p* = 0.007), *p_Actinobacteriota* (*p* = 0.036), and *p_Cyanobacteria* (*p* = 0.022) (Figure 4C–E). These phyla are involved in degrading complex carbohydrates, promoting short-chain fatty acid production, maintaining intestinal barrier integrity, and suppressing intestinal inflammation. In contrast, GG-PPC significantly decreased the abundance of potentially harmful phyla, including *p_Firmicutes* (*p* = 0.024), *p_Campilobacterota* (*p* = 0.050), and *p_Desulfobacterota* (Figure 4F–H). A reduced Firmicutes/Bacteroidota ratio (Figure S1A) helped alleviate metabolic disorders and lowered the risk of pathogenic colonization and intestinal inflammation. Conversely, XG-PPC had milder regulatory effects, significantly increasing the relative abundances of *p_Bacteroidota* (*p* = 0.007), but showed less pronounced modulation of most other beneficial and harmful phyla.

At the genus level (Figure 4B), XG-PPC and GG-PPC differentially modulated the relative abundance of several key gut microbial genera compared with that of the Control group. The relative abundances of *Bacteroidota*-associated genera, including *g_Muribaculaceae* (XG-PPC: *p* = 0.017; GG-PPC: *p* = 0.006), *g_Bacteroides* (XG-PPC: not significant; GG-PPC: *p* = 0.022), and *g_Alistipes* (XG-PPC: *p* = 0.003; GG-PPC: *p* = 0.007), increased in both the treatment groups, with greater enrichment in the GG-PPC group (Figure 4I–K). These genera are commonly associated with carbohydrate metabolism and short-chain fatty acid production and are considered important contributors to intestinal homeostasis and inflammation alleviation. Notably, *g_Alloprevotella* (*p* = 0.026) was significantly enriched only in the XG-PPC group, suggesting a differential utilization pattern of the two polysaccharides by specific microbial taxa (Figure S1B). The relative abundance of *g_Lactobacillus* (XG-PPC: *p* = 0.001; GG-PPC: *p* < 0.001) was significantly reduced after treatment with both XG-PPC and GG-PPC (Figure 4M), consistent with the overall decrease in *Firmicutes* abundance. This shift may contribute to changes in the *Firmicutes*/*Bacteroidetes* balance and improve the host metabolic microenvironment after polysaccharide intervention. In contrast, as shown in Figure S1C, *g_Lachnospiraceae* (*p* = 0.008) was significantly enriched only in the XG-PPC group, indicating the distinct modulatory effects of the two polysaccharides on specific *Firmicutes*-associated taxa. Regarding potentially pathogenic bacterial genera, the abundance of *g_Helicobacter* (*p* = 0.050) was significantly reduced in the GG-PPC group (Figure 4N), suggesting that GG-PPC may suppress the proliferation of inflammation-associated microorganisms. Within the phylum *Proteobacteria*, *g_Enterorhabdus* (XG-PPC: *p* = 0.005; GG-PPC: *p* = 0.001) exhibited varying degrees of enrichment in the polysaccharide intervention groups (Figure S1E,D), which is hypothesized to represent an adaptive compensatory change during the comprehensive structural reorganization of the gut microbiota. In addition, *g_Akkermansia* exhibited a greater enrichment trend in the GG-PPC group as shown in Figure 4L. Given its recognized association with mucus layer colonization and intestinal barrier maintenance, this finding suggests a potential role for GG-PPC in supporting the gut barrier function.

**Figure 5:**
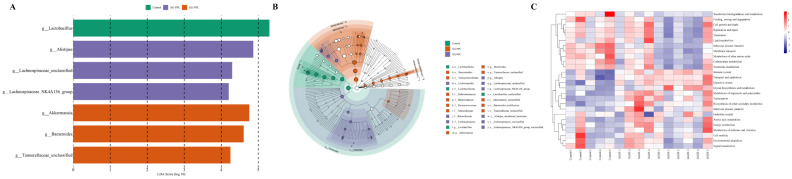
(**A**) LEfSe differential species analysis (LDA threshold > 3.0) was performed to identify significantly enriched bacterial taxa between groups, (**B**) Cladogram of differential species, illustrating evolutionary distribution characteristics spanning from the phylum to the genus level, (**C**) Heatmap of KEGG functional pathways, in which red denotes increased pathway abundance and blue denotes decreased levels.

LEfSe analysis, using a Linear Discriminant Analysis (LDA) score threshold of >4, was used to identify differentially abundant microbial taxa among the three groups. The top 20 differential features ranked by the LDA score were selected for visualization and further analysis (Figure 5A,B). The results revealed significant differences in gut microbial composition across multiple taxonomic levels among the Control, GG-PPC, and XG-PPC groups (*p* < 0.05). The characteristic taxa in the control group were predominantly affiliated with the phylum *Firmicutes*, including *g_Lactobacillus* and its associated taxonomic levels, such as *f_Lactobacillaceae*, *o_Lactobacillales*, and *c_Bacilli*, with the highest LDA score (5.32). These findings indicate that *Lactobacillus*-related taxa are the principal microbial signatures in the Control group. The differential taxa in the GG-PPC group were mainly enriched within the phyla *Bacteroidetes* and *Verrucomicrobia*, with *g_Bacteroides*, *f_Bacteroidaceae*, and *p_Bacteroidota* being significantly enriched. In addition, *g_Akkermansia* and its associated taxa, including *f_Akkermansiaceae* and *p_Verrucomicrobiota*, were identified as key discriminatory biomarkers of the GG-PPC group. Furthermore, *f_Ruminococcaceae*, *f_Tannerellaceae*, and their unclassified genera were specifically enriched in the GG-PPC group, suggesting that dried shiitake mushroom polysaccharides significantly drove the proliferation of these genera, making them core targets for regulating the gut microbiota. The differential taxa in XG-PPC were primarily associated with the *Clostridia* class of *Firmicutes*, including *g_Alistipes*, *f_Rikenellaceae*, *o_Lachnospirales*, *f_Lachnospiraceae*, their unclassified genera, and the *NK4A136_group*, with LDA scores ranging from 4.07 to 4.85. This indicates that XG-PPC tends to enrich genera related to *Lachnospiraceae* and *Rikenellaceae*, demonstrating a distinct pattern of microbiota regulation compared with GG-PPC. PICRUSt (Picrust2, 2.5.2) functional prediction analysis (Figure 5C) was used to investigate the differential enrichment of KEGG functional pathways in the gut microbiota of mice in the different treatment groups. Compared with those in the Control group, the functional pathway profiles of the gut microbiota were significantly altered after XG-PPC and GG-PPC interventions, with distinct differences in enrichment patterns. The XG-PPC group exhibited a unique profile characterized by significant upregulation of core metabolic pathways, including those involved in amino acid metabolism, energy production, and cofactor and vitamin synthesis. Concurrently, pathways related to bacterial and parasitic diseases were significantly enriched. These findings suggest that XG-PPC substantially enhances the basal metabolic capacity of the gut microbiota, although specific functional compensatory changes associated with pathogens occur during the microbial restructuring. Overall, these results confirm that GG-PPC effectively modulates the gut microbiome by targeting its metabolic and immunomodulatory activities, providing a functional basis for elucidating the structure-dependent probiotic mechanisms of Biyang *flower shiitake mushroom* macromolecules.

### 3.7. Exploration of Differentially Abundant Metabolites Modulated by XG-PPC and GG-PPC Using Untargeted Fecal Metabolomics

Untargeted metabolomic analysis of fecal samples from the Control, XG-PPC, and GG-PPC groups was performed to elucidate the metabolic changes induced by different Biyang *flower shiitake mushroom* macromolecular treatments.

**Figure 6:**
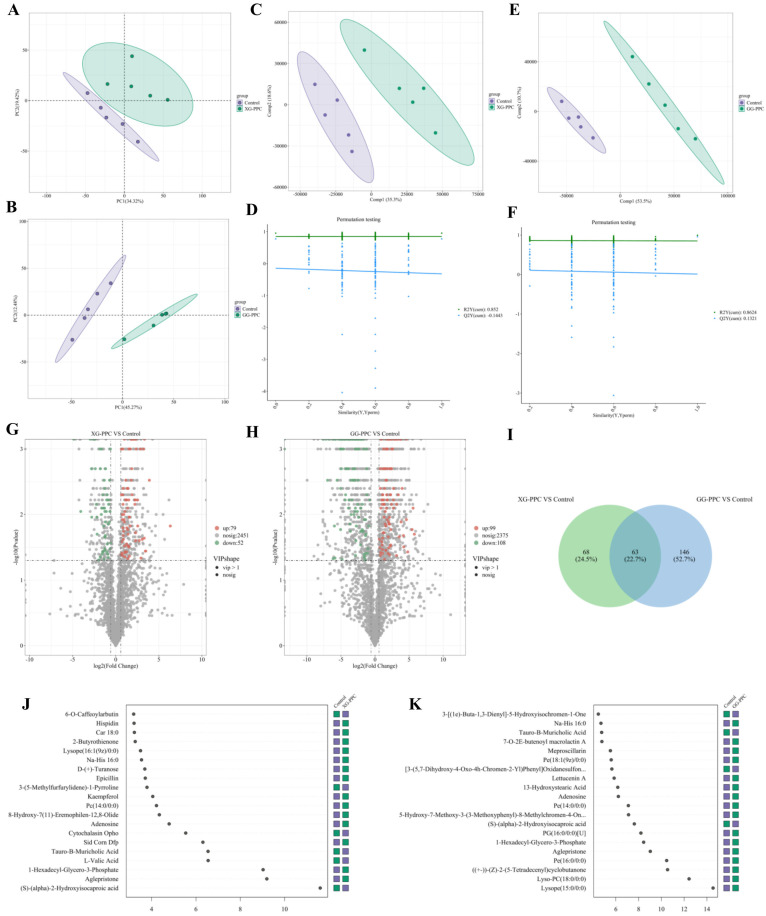
Metabonomic analysis of fecal samples (*n* = 5) (**A**,**B**) PCA score plots between Control and XG-PPC (**A**), and between Control and GG-PPC (**B**), (**C**–**F**) OPLS-DA and relative permutation tests of between the Control and XG-PPC groups (**C**,**D**), and between the Control and GG-PPC groups (**E**,**F**), (**G**,**H**) Volcano plots of differential metabolites in the comparisons of (**G**) XG-PPC vs. Control and (**H**) GG-PPC vs. Control. Red indicates significantly upregulated metabolites, blue indicates significantly downregulated metabolites, and gray indicates non-significant metabolites (VIP > 1, *p* < 0.05). (**I**) Venn diagram illustrating the distribution of common and specific differential metabolites. (**J**,**K**) Top 20 key differential metabolites sorted by VIP values in (**J**) XG-PPC vs. Control and (**K**) GG-PPC vs. Control groups.

After quality control, mass spectrometric data validation in both positive and negative ion modes, metabolite annotation, and classification analysis, 2849 metabolites were identified (Figure S2A,B). As shown in Figure 6B, PCA revealed a distinct inter-group separation between the Control and GG-PPC groups in the score plot. PC1 and PC2 accounted for 45.27% and 12.48% of the total metabolic variance, respectively. The tight intra-group clustering of the biological replicates within each group suggested good experimental reproducibility. In contrast, the XG-PPC group exhibited partial separation from the Control group (Figure 6A), indicating greater metabolic heterogeneity within this group. Partial Least Squares Discriminant Analysis (PLS-DA) models were validated by permutation testing (GG-PPC group: R^2^Y = 0.8624, Q^2^Y = 0.1321; XG-PPC group: R^2^Y = 0.852, Q^2^Y = −0.1443), indicating acceptable model stability and a low risk of overfitting (Figure 6C–F). The PLS-DA results further supported the distinct metabolic profiles observed among the treatment groups. Differentially expressed metabolites (DEMs) were identified using criteria of |Log_2_FC| > 1.5, *p* < 0.05, and variable importance for the projection (VIP) > 1. Volcano plot analysis (Figure 6H) identified 207 DEMs in the GG-PPC group relative to the control group, including 99 upregulated and 108 downregulated DEMs (Table S2). In the XG-PPC group, 131 DEMs were identified, comprising 79 upregulated and 52 downregulated DEMs (Figure 6G, Table S1). As shown in Figure 6I, the Venn diagram analysis revealed clear differences in the composition of the DEMs between the two treatment groups. Specifically, 146 unique DEMs were identified in the GG-PPC group, accounting for 52.7% of the total, suggesting that GG-PPC induced broader metabolic alterations than XG-PPC. As shown in Figure 6K, in the comparison between the Control and GG-PPC groups, DEMs with higher VIP scores primarily include bile acids (e.g., Tauro-B-Muricholic Acid), lipids and phospholipids (e.g., Lyso-PC (18:0/0:0), 1-hexadecyl-glycero-3-phosphate), and nucleotides (e.g., Adenosine). In contrast, DEMs with higher VIP scores in the control versus XG-PPC comparison mainly included amino acid derivatives (e.g., L-valic acid), lipids, and secondary plant metabolites (e.g., Kaempferol). These metabolites are potentially involved in biological processes related to gut microbial metabolism, bile acid homeostasis, energy metabolism, and inflammation.

**Figure 7:**
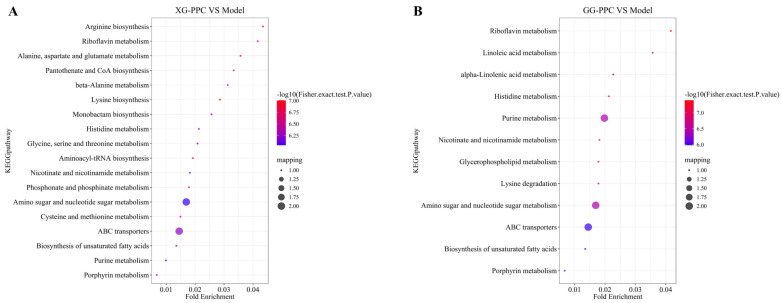
KEGG enrichment analysis of differential metabolites between (**A**) Control and XG-PPC, and (**B**) between Control and GG-PPC.

KEGG pathway enrichment analysis of the DEMs revealed that both XG-PPC and GG-PPC interventions significantly modulated biological pathways of fecal metabolites in mice, with each exhibiting distinct enrichment profiles. GG-PPC notably enriched several key metabolic pathways (Figure 7B), including purine, lysine, and histidine metabolism; amino sugar and nucleotide sugar metabolism; glycerophospholipid metabolism; linoleic acid metabolism; α-linolenic acid metabolism; riboflavin metabolism; nicotinate and nicotinamide metabolism; porphyrin metabolism; biosynthesis of unsaturated fatty acids; and ABC transporters. In contrast, the XG-PPC intervention primarily enriched pathways (Figure 7A), associated with arginine biosynthesis, purine metabolism, alanine, aspartate, and glutamate metabolism, glycine, serine, and threonine metabolism, β-alanine metabolism, amino sugar and nucleotide sugar metabolism, riboflavin metabolism, porphyrin metabolism, biosynthesis of unsaturated fatty acids, and ABC transporters, highlighting a greater association with amino acid, nucleotide, and carbohydrate metabolism. Collectively, these findings indicate that GG-PPC and XG-PPC differentially influence host metabolic pathways, with GG-PPC showing stronger associations with lipid, fatty acid, and vitamin metabolism and XG-DT being more closely associated with amino acid and nucleotide metabolism. These results suggest that drying treatment may alter the metabolic responses elicited by Biyang *flower shiitake mushroom* macromolecules, potentially contributing to differences in gut microbial and metabolic homeostasis by modulating various metabolic pathways.

### 3.8. Correlations Among Physicochemical Properties, Gut Microbiota, and Differential Metabolites of Biyang Flower Shiitake Mushroom Macromolecules

**Figure 8:**
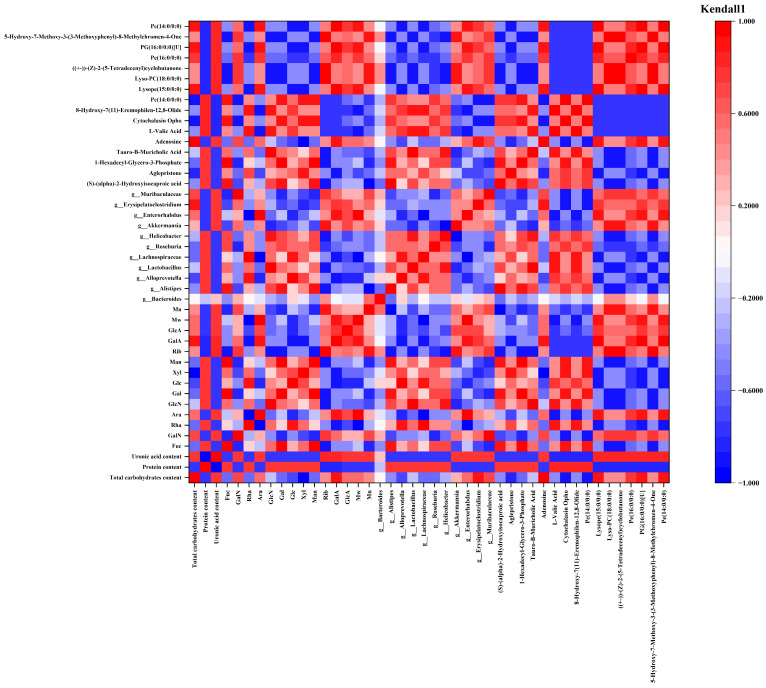
Kendall correlation analysis between differential metabolites, gut microbiota, and polysaccharide-related chemical components.

Kendall’s rank correlation analysis revealed that the physicochemical differences between XG-PPC and GG-PPC were closely associated with their distinct modulatory effects on the gut microbiota and metabolome (Figure 8).

Correlation analysis between macromolecular structure and gut microbiota (Table S3) revealed that the relationship between protein and uronic acid contents was the most prominent. Specifically, protein content exhibited a strong positive correlation with six bacterial genera, including *g_Alistipes* and *g_Lactobacillus*, and a strong negative correlation with four bacterial genera, such as *g_Akkermansia* and *g_Muribaculaceae*. At the metabolite level, protein content was positively and negatively associated with non-lipid and lipid metabolites, respectively. In contrast, uronic acid content exhibited an entirely opposite correlation pattern. This suggests that the levels of protein and uronic acid content mediate their biological effects by regulating distinct gut microbiota and metabolic pathways. Total carbohydrate content showed a significant negative correlation with *g_Alloprevotella*, *g_Aglepristone*, and the metabolites Cytochalasin Opho and Pc (14:0/0:0). In contrast, total carbohydrate content exhibited significant positive correlations with two genera (*g_Erysipelatoclostridium* and *g_Muribaculaceae*), and multiple lipid metabolites (e.g., Lysope (15:0/0:0), Pe (16:0/0:0)).

Glc is the dominant monosaccharide constituent common to XG-PPC and GG-PPC. Kendall correlation analysis showed that Glc content was significantly positively correlated with the abundance of *g_Lachnospiraceae* and the levels of L-Valic Acid and 8-Hydroxy-7(11)-Eremophilen-12,8-Olide, while significantly negatively correlated with the abundance of *g_Enterorhabdus*, and the levels of Adenosine and various lipid metabolites (e.g., Lysope (15:0/0:0), Pe (16:0/0:0)). Man and Gal are minor monosaccharide components of two types of polysaccharide complexes, and their regulatory patterns on the gut microbiota and metabolites are highly consistent. Correlation analysis revealed showed that their contcentrations were significantly positively correlated with *g_Alistipes*, *g_Roseburia*, *g_Helicobacter*, (S)-(α)-2-Hydroxyisocaproic acid, Cytochalasin Opho, and Pc (14:0/0:0), and significantly negatively correlated with *g_Akkermansia*, *g_Erysipelatoclostridium*, *g_Muribaculaceae*, Lyso-PC (18:0/0:0), (±)-(Z)-2-(5-Tetradecenyl) cyclobutanone, and 5-Hydroxy-7-Methoxy-3-(3-Methoxyphenyl)-8-Methylchromen-4-One. Rib and Gal A are characteristic monosaccharide components that distinguish GG-PPC from XG-PPC. Correlation analysis showed that Rib content was significantly positively correlated with the mucin-degrading bacteria *g_Akkermansia* and *g_Muribaculaceae*, while it was significantly negatively correlated with gut genera including *g_Alistipes*, *g_Roseburia*, and *g_Helicobacter*, as well as with multiple metabolites such as amino acids and branched-chain organic a e.g.,cid (L-Valic Acid, (S)-α-2-Hydroxyisocaproic acid), bile acid metabolites (e.g., Tauro-β-Muricholic Acid), and glycerophospholipid (e.g., 1-Hexadecyl-Glycero-3-Phosphate, Pc (14:0/0:0)). Compared with GG-PPC, XG-PPC contains higher levels of Rha and Fuc. Kendall correlation analysis reveals that Fuc is significantly positively correlated with *g_Alistipes*, *g_Roseburia*, and a variety of small-molecule metabolites, whereas it demonstrates significant negative correlations with two beneficial mucin-degrading bacteria (*g_Akkermansia* and *g_Muribaculaceae*), as well as diverse lipid and flavonoid metabolites. Similar to Fuc, Rha increases the relative abundance of *g_Alloprevotella*, and both monosaccharides suppress the production of multiple lipid metabolites.

Mn and Mw exhibited weak overall correlations with differential intestinal bacterial genera, and their regulatory effects were largely mediated through intestinal metabolites. Both Mn and Mw showed significant negative correlations with g_Aglepristone and various metabolites, including amino acid metabolites, bile acids, glycerophospholipids, and terpenoids. In contrast, Mn and Mw exhibited significant positive correlations with lipid components (e.g., Lysope (15:0/0:0), Pe (16:0/0:0)) and flavonoid derivatives exemplified by 5-Hydroxy-7-Methoxy-3-(3-Methoxyphenyl)-8-Methylchromen-4-One. Collectively, these findings suggest that XG-PPC and GG-PPC demonstrated distinct regulatory patterns on the intestinal microbiota and metabolites.

## 4. Discussion

This study systematically characterized the physicochemical and structural properties of XG-PPC and GG-PPC, *macromolecules* isolated from Biyang *flower shiitake mushroom*. The differential regulatory effects of these macromolecules on the gut microbiota and fecal metabolome of mice were evaluated, and the interrelationships among macromolecules’ physicochemical characteristics, key intestinal bacterial genera, and differential metabolites were elucidated using Kendall rank correlation analysis. This approach provides insights into the association between macromolecules’ structural characteristics and variations in the gut microbiota composition and metabolic profiles.

Previous studies have indicated that Glc-rich fungal heteroglycans can increase the abundance of beneficial bacteria such as *Bacteroidetes* and *Akkermansia*, decrease the Firmicutes/Bacteroidetes ratio, and improve gut homeostasis [39,40,41]. In this study, both XG-PPC and GG-PPC mirrored this classic regulatory phenotype, confirming the broad-spectrum gut-protective effect of edible fungal polysaccharides. Edible fungi are abundant sources of polysaccharides and proteins. However, previous studies have predominantly investigated on single-component polysaccharides or proteins from edible fungi [42,43,44], while few studies have compared two types of structurally similar but compositionally distinct polysaccharide-protein complexes derived from the same fungus. Consequently, elucidating how structurally analogous macromolecules exhibiting subtle variations reshape the gut metabolic profile remains challenging. In this study, although XG-PPC and GG-PPC exhibited highly similar profiles of major monosaccharide and backbone structures, the unique presence of ribose and galacturonic acid, combined with a higher polydispersity index and a loose ellipsoidal microstructure, endowed GG-PPC with stronger prebiotic effects. Specifically, GG-PPC simultaneously regulated multiple lipid and vitamin metabolic pathways, and its effect on promoting homogeneity within the gut microbiota community was more stable. In contrast, XG-PPC, characterized by a higher Glc content and severe molecular aggregation, selectively enriched the *Lachnospiraceae* family while predominantly disrupting amino acid and nucleotide metabolism. These findings complement the existing structure-activity relationship framework, indicating that acidic sugar composition, molecular polydispersity, and microstructure are key fine structural features that determine the gut regulatory targets of edible fungal macromolecules, and that sole reliance on the composition of major monosaccharides is insufficient to accurately predict their in vivo metabolic regulatory specificities.

Notably, existing literature frequently posits that molecular weight is a primary determinant of the bioavailability of macromolecules [45]. However, the correlation analysis in this study revealed divergent findings: weight-average and number-average molecular weights exhibited no significant direct correlation with gut microbiota abundance, but may instead influence these parameters via the indirect modulation of metabolic pathways, including those involving bile acids and glycerophospholipids.

Our study has several limitations. First, the tested macromolecules were polysaccharide–protein complexes, and the observed gut-modulating effects reflect the combined action of both components. As separate evaluations of the purified polysaccharide and protein fractions were not conducted, the core bioactive moiety remains undefined. Second, microbial functional profiles were predicted solely using PICRUSt, without validation by metatranscriptomic or metaproteomic approaches, which fails to capture the dynamic transcriptional and translational activities of intestinal microbes in vivo. Finally, this study was limited to Kendall rank correlation analysis for examining associations among macromolecular structural indicators, gut microbiota, and metabolites. While this method can identify statistical correlations between variables, it is insufficient to fully elucidate the complex cascade mechanism through which macromolecules regulate the gut microbiota via stepwise transduction pathways.

## 5. Conclusions

In conclusion, this study, by integrating multi-omics approaches with structural characterization, demonstrated that the distinct gut modulation patterns of two polysaccharide-protein complexes (XG-PPC and GG-PPC) from Biyang *flower shiitake mushroom* stem from differences in molecular dispersity, acidic sugar composition, and micro-scale aggregation states. Specifically, GG-PPC (low dispersity, containing galacturonic acid and ribose) stably remodeled the gut microbiota while concomitantly modulating lipid and vitamin metabolism, whereas XG-PPC (high glucose, particle-aggregated) selectively enriched short-chain fatty acid (SCFA)-producing bacteria and primarily disrupted amino acid and nucleotide metabolism. Correlation analysis further identified uronic acid and protein as core structural factors, with molecular weight exerting only an indirect influence on metabolic pathways. This study investigated the structure-activity relationships of macromolecules from Biyang *flower shiitake mushroom*, establishing a theoretical foundation for the high-value utilization of this edible fungus and the targeted development of functional foods for gastrointestinal health.

## Figures and Tables

**Table 1:** Yield, chemical composition, molecular weight and monosaccharide composition of XG-PPC and GG-PPC.

Item	XG-PPC	GG-PPC
Yield (%)	0.81	2.13
**Chemical characteristics**		
Carbohydrate (%)	31.56	34.8
Protein (%)	37.99	35.46
Uronic acid (%)	4.27	6.9
**Monosaccharide composition (** **μ** **g/mg)**		
Fuc	6.485	3.146
GalN	0.804	1.198
Rha	4.379	2.375
Ara	0.592	1.024
GlcN	6.732	6.26
Gal	68.625	37.538
Glc	526.913	314.345
Xyl	0.726	0.498
Man	32.252	24.327
Rib	0	5.446
GalA	0	2.021
GlcA	7.501	8.135
**Molecular weight (kDa)**		
**M_w_**	3.186 × 10^5^	3.891 × 10^5^
**M_n_**	2.021 × 10^5^	4.097 × 10^4^
**M_w_/M_n_**	1.576	9.496

## Data Availability

The original contributions presented in this study are included in the article and Supplementary Materials. Further inquiries can be directed to the corresponding author.

## Supplementary Materials

The following supporting information can be downloaded at: https://www.mdpi.com/article/10.3390/nu18142289/s1, Figure S1: Alterations in F/B ratio and relative abundance of characteristic gut bacterial taxa after XG-PPC and GG-PPC intervention. (A) The ratio of Firmicutes to Bacteroidetes (F/B ratio). (B–E) Relative abundance of *g_Alloprevotella*, *g_Lachnospiraceae*, *g_Enterorhabdus*, and *p_Proteobacteria*, respectively. Values are expressed as mean ± SD; ns denotes no significant difference.; Figure S2: Total ion chromatograms (TICs) of untargeted metabolomics profiling for fecal samples under positive and negative ion modes. (A) Positive ion (POS) mode; (B) Negative ion (NEG) mode; Table S1: Detailed information of differential metabolites (|Log2FC| > 1.5, *p* < 0.05, and VIP > 1) between XG-PPC and Control groups; Table S2: Detailed information of differential metabolites (|Log2FC| > 1.5, *p* < 0.05, and VIP > 1) between GG-PPC and Control groups; Table S3: Matrix for correlation analysis.
