# Supplementary material for: Physicochemical Properties and Gut Microbiota-Regulating Activities of Macromolecules from Fresh and Dried Biyang Flower Shiitake Mushrooms: A Comparative Study Integrating 16S rRNA Sequencing and Untargeted Metabolomics
Source: Nutrients. 2026 Jul 13;18(14):2289. doi: 10.3390/nu18142289 (PMC13415118; doi:10.3390/nu18142289)
Supplement: Supplementary file 1 [file nutrients-18-02289-s001.zip › nutrients-4398951-supplementary.pdf]

Physicochemical Properties and Gut Microbiota-Regulating Activities of Macromolecules from Fresh and Dried Biyang *Flower Shiitake*: A Comparative Study Integrating 16S RRNA Sequencing and Untargeted Metabolomics

Shunchao Zhang[1], Xiling Fan[2], Xinli Wei[3] and Kai Li [2*]

[1] School of Management, Henan University of Chinese Medicine, Zhengzhou 450046, China;

[2] College of Pharmacy, Henan University of Chinese Medicine, Zhengzhou 450046, China;

[3] The First Affiliated Hospital of Henan University of Chinese Medicine, Zhengzhou 450046, China;

Fig. S1. Alterations in F/B ratio and relative abundance of characteristic gut bacterial taxa after XG-PPC and GG-PPC intervention. (A) The ratio of Firmicutes to Bacteroidetes (F/B ratio). (B–E) Relative abundance of *g_Alloprevotella*, *g_Lachnospiraceae*, *g_Enterorhabdus*, and *p_Proteobacteria*, respectively. Values are expressed as mean ± SD; ns denotes no significant difference.

Fig. S2. Total ion chromatograms (TICs) of untargeted metabolomics profiling for fecal samples under positive and negative ion modes. (A) Positive ion (POS) mode; (B) Negative ion (NEG) mode.

Table S1. Detailed information of differential metabolites ($|Log_2FC| > 1.5$, $P < 0.05$, and VIP > 1) between XG-PPC and Control groups.

Table S2. Detailed information of differential metabolites ($|Log_2FC| > 1.5$, $P < 0.05$, and VIP > 1) between GG-PPC and Control groups.

Table S3. Matrix for correlation analysis.

Fig. S1. Alterations in F/B ratio and relative abundance of characteristic gut bacterial taxa after XG-PPC and GG-PPC intervention. (A) The ratio of Firmicutes to Bacteroidetes (F/B ratio). (B–E) Relative abundance of *g_Alloprevotella*, *g_Lachnospiraceae*, *g_Enterorhabdus*, and *p_Proteobacteria*, respectively. Values are expressed as mean ± SD; ns denotes no significant difference.

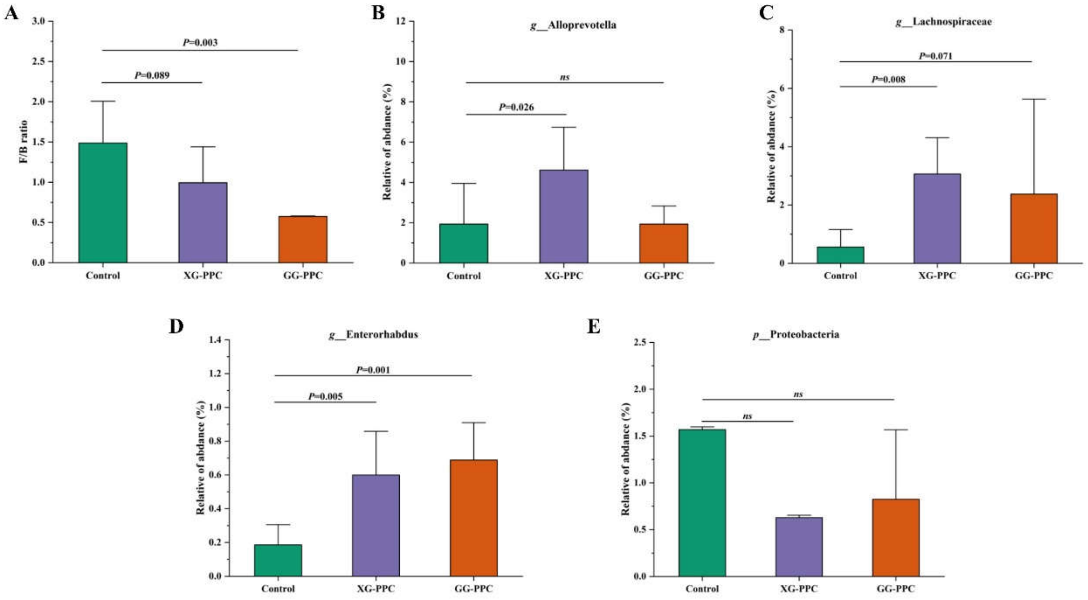

Fig. S2. Total ion chromatograms (TICs) of untargeted metabolomics profiling for fecal samples under positive and negative ion modes. (A) Positive ion (POS) mode; (B) Negative ion (NEG) mode.

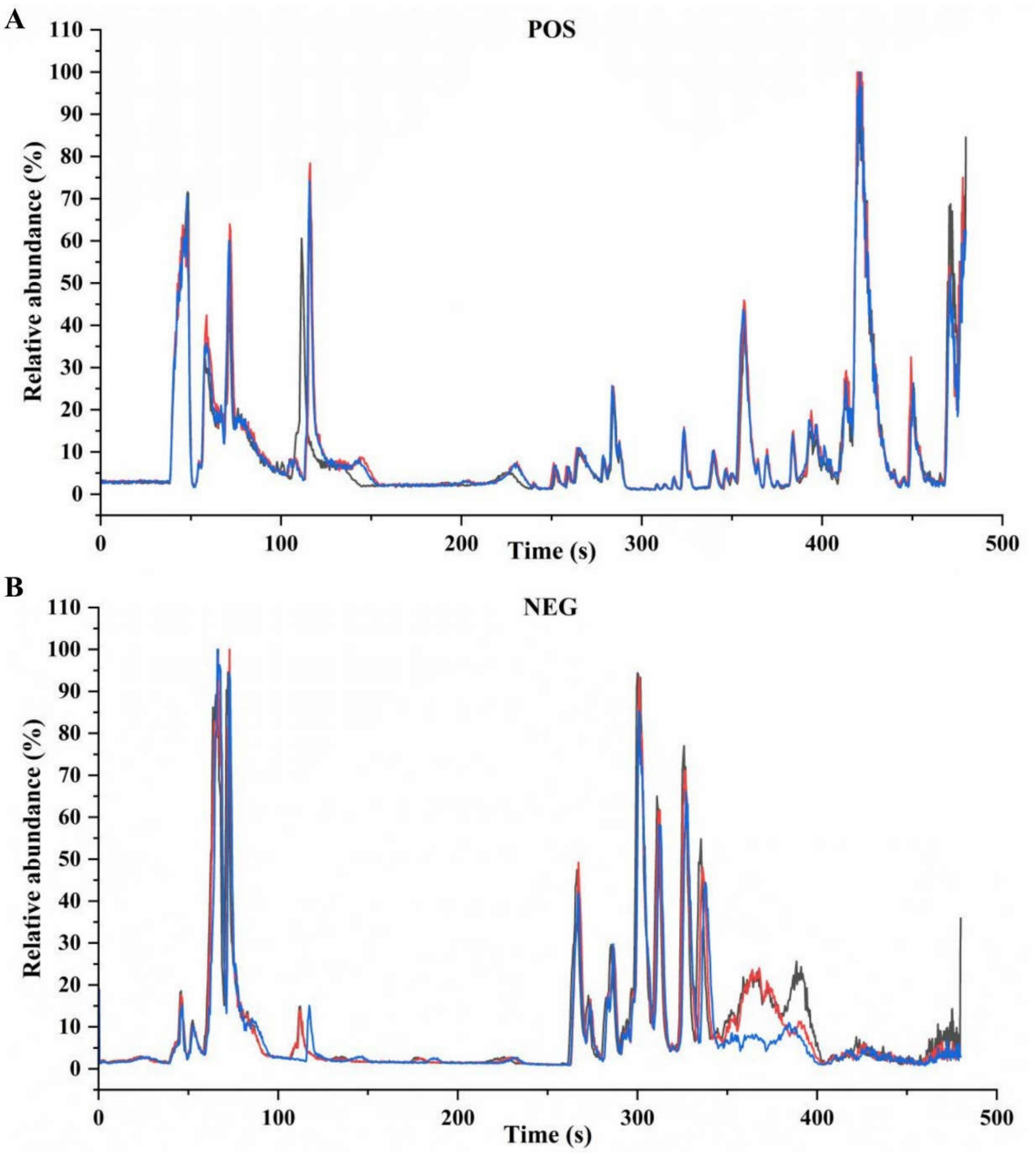

Table S1. Detailed information of differential metabolites ($|Log_2FC| > 1.5$, $P < 0.05$, and VIP > 1) between XG-PPC and Control groups.

Metabolite	FC	VIP	P value	Sig
Arabinose	1.621	1.371	0.026	up
N-Acetyl-Mannosamine	1.562	1.401	0.001	up
3-Indoleglyoxylic Acid	1.961	1.594	0.012	up
A-Hydroxylevulinic Acid	1.638	1.312	0.039	up
[(12-Methyltetraphen-7-Yl)Methoxy]Sulfonic Acid	3.236	1.299	0.001	up
Diacetoxy-6-Gingerdio	80.580	1.435	0.015	up
Prelactone B	1.753	1.167	0.005	up
Hexylitaconic Acid	1.831	1.012	0.004	up
Alpha-D-Ribose 1-Methylphosphonate 5-Phosphate	9.319	1.986	0.001	up
3-Oxocapric Acid	1.774	1.044	0.001	up
5,7-Dimethoxyflavanone	3.483	1.061	0.003	up
3b-Hydroxy-6b-Tigloyloxy-7(11)-Eremophilen-12,8b-Olide	5.180	1.207	0.001	up
6-{2,5-dihydroxy-3-[(E)-2-(4-hydroxyphenyl)ethenyl]-6-[(1E)-3-methylbut-1-en-1-yl]phenoxy}-3,4,5-trihydroxyoxane-2-carboxylic acid	4.755	2.196	0.006	up
3,8-Dihydroxycoumarin	2.141	1.125	0.037	up
2-Butyrothienone	4.559	3.261	0.000	up
Kaempferol	3.631	4.053	0.002	up
Eriodictyol	7.285	1.939	0.001	up
Hydroxy Ibuprofen	3.771	1.815	0.000	up
4l4v	1.802	1.291	0.007	up
Absintholide	1.710	2.430	0.042	up
24,25-Epoxywithanolide D	1.578	2.083	0.005	up
N4 2'-O-Dibutyrylcytidine 3'	4.056	2.720	0.000	up
11b-Hydroxyprogesterone	3.530	1.698	0.000	up
Trh-Oh	6.597	1.453	0.025	up
Mercapturic Acid	8.409	1.594	0.024	up
Glycerol 1-Propanoate Diacetate	10.939	1.125	0.036	up
Dide-O-Methylsimmondsin	11.127	1.875	0.036	up
5b-Cyprinol Sulfate	2.594	1.154	0.008	up
Armexifolin	3.591	1.530	0.000	up

8-Hydroxy-7(11)-Eremophilen-12,8-Olide	3.668	4.349	0.000	up
Pa(22:4(7z,10z,13z,16z)/0:0)	2.378	1.378	0.028	up
Lpe 18:1	2.684	1.538	0.020	up
GPGro(2:0/18:2)	4.661	1.454	0.007	up
GPGro(2:0/18:1)	2.854	2.979	0.006	up
D-(+)-Turanose	2.810	3.694	0.030	up
1-Kestose	2.651	1.750	0.047	up
Glucosamine 1-Phosphate	2.631	1.344	0.017	up
Galbeta1,3glcnac	2.429	3.173	0.016	up
Epicillin	2.386	3.716	0.037	up
Adenosine	1.528	4.792	0.041	up
Leucozenenol	14.927	1.401	0.003	up
Linagliptin	4.916	1.103	0.025	up
3,6-Ditigloyloxytropan-7-Ol	5.868	1.285	0.015	up
Blue Acid	10.040	2.044	0.000	up
N-Eicosapentaenoyl Tyrosine	2.033	2.001	0.030	up
Hydrocortisone Phosphate	3.021	1.094	0.011	up
Gentamicin C1a	2.506	1.161	0.004	up
N-Arachidonoyl Phenylalanine	3.719	1.061	0.005	up
Acrl Toxin Ii	7.589	1.604	0.046	up
N-Docosahexaenoyl Valine	2.391	2.489	0.006	up
1,4-Diacetoxypalitantin	2.995	1.624	0.043	up
Sid Corn Dfp	2.687	6.314	0.004	up
Hispidin	3.193	3.209	0.015	up
Pfizer 105696,Cp105696, Cp-105696, Cp 105,696,Cp-105,696,Cp105,696	1.896	1.086	0.018	up
4-Oleamidobutanoic Acid	1.853	1.678	0.021	up
Procurcumenol	3.406	1.028	0.000	up
1z1h	2.249	2.087	0.003	up
N-(2-oxo-2-piperazin-1-ylethyl)-N-[3-(2-oxopyrrolidin-1-yl)propyl]-4-(trifluoromethyl)benzamide	4.920	1.328	0.019	up
3'-C-Ethynylcytidine	5.783	1.140	0.006	up
Ganoderiol A	1.996	1.434	0.013	up
Hydroxygaleon	9.861	1.454	0.023	up
Psychosine From Bovine Brain	1.854	1.109	0.024	up
Oocyan	2.901	1.364	0.031	up
Pc(14:0/0:0)	2.010	4.220	0.000	up
(2r,4bs,6as,12bs,12cr,14as)-2'-Hydroxypaxilline	2.678	1.854	0.030	up
Pe(22:6(4z,7z,11e,13z,15e,19z)-2oh(10s,17)/16:0)	4.671	1.216	0.006	up

Lpa(16:0/0:0)	1.724	1.268	0.012	up
Lysope(16:1(9z)/0:0)	1.907	3.500	0.010	up
Heliannuol D	5.121	1.277	0.000	up
Pa(17:0/0:0)	1.663	1.174	0.010	up
Eicosanoyl-Ea	2.041	1.732	0.038	up
1-Hexadecyl-Glycero-3-Phosphate	1.776	9.030	0.016	up
Aglepristone	2.372	9.198	0.006	up
Car 18:0	2.879	3.223	0.001	up
LPA(i-19:0/0:0)	2.206	1.964	0.006	up
Me-Tpa	2.953	1.472	0.011	up
Na-His 16:0	2.087	3.546	0.044	up
Butylamine	2.095	1.287	0.009	up
(3z)-Phycocyanobilin	5.050	1.366	0.000	up
Aspartic Acid	0.431	1.071	0.013	down
Ribavirin Monophosphate	0.218	1.597	0.011	down
{[2,3,4-Trihydroxy-5-(Hydroxymethyl)Oxolan-2-Yl]Methoxy}Sulfonic Acid	0.413	1.172	0.004	down
N-acetyl-S-(2-succino)-L-cysteine	0.145	2.149	0.011	down
Acetonic Acid	0.549	1.047	0.045	down
Tyrosyl-Glycine	0.379	1.380	0.008	down
5-Methoxycarbonyl-2-Thiophenecarboxylic Acid	0.504	1.770	0.005	down
L-Valic Acid	0.172	6.551	0.000	down
Gamma-Glutamylleucine	0.194	2.313	0.002	down
Fema No. 3941	0.442	1.552	0.002	down
17-Phenyl-18,19,20-Trinor-Prostaglandin D2	0.355	1.861	0.037	down
Alaptide	0.424	1.117	0.022	down
Leucic Acid	0.034	1.426	0.000	down
(S)-(alpha)-2-Hydroxyisocaproic acid	0.129	11.606	0.000	down
6-O-Caffeoylarbutin	0.565	3.192	0.006	down
N-Palmitoyl Aspartic acid	0.287	1.429	0.044	down
Ergosecaline	0.142	1.064	0.002	down
Sulfolithocholylglycine	0.224	2.478	0.020	down
Asperterpene H	0.323	1.682	0.008	down
Cytochalasin Opho	0.210	5.540	0.007	down
6-Gingesulfonic Acid	0.280	1.924	0.001	down
(R)-Equol	0.400	2.127	0.003	down
Enterolakton	0.319	1.318	0.001	down
Leu-Arg-Asn-Arg	0.407	1.274	0.008	down
(2s,3s,5s,8r,9s,10s,13s,14s,16s,17r)-17-Acetyloxy-10,13-Dimethyl-2-	0.338	1.453	0.040	down

Morpholin-4-Yl-16-(1-Prop-2-Enylpyrrolidin-1-Ium-1-Yl)-2,3,4,5,6,7,8,9,11,12,14,15,16,17-Tetradecahydro-1h-Cyclopenta[A]Phenanthren-3-Olate (8r,9s,10s,13s,14s,17s)-17-Hydroxy-10,13-Dimethyl-4,5,6,7,8,9,11,12,14,15,16,17-Dodecahydro-1h-Cyclopenta[A]Phenanthrene-2,3-Dione	0.561	1.358	0.007	down
Cervonic Acid	0.638	1.543	0.033	down
Zoledronic Acid	0.480	1.375	0.006	down
Cephalotin Acid	0.188	1.122	0.000	down
Asn Leu	0.630	2.421	0.009	down
Aconine	0.511	1.910	0.028	down
2-Hydroxyacorenone	0.259	1.437	0.049	down
Isofloxythepin	0.349	3.182	0.037	down
6-O-Oleuropeoylsucrose	0.411	1.477	0.033	down
5-Hpq	0.486	2.061	0.037	down
3-(5-Methylfurfurylidene)-1-Pyrroline	0.060	3.792	0.009	down
N-(Lysergyl-Isoleucyl)-Cyclo(Phenylalanyl-Prolyl)	0.314	1.061	0.010	down
Isoleucylleucine	0.659	2.758	0.027	down
Arg-Arg-Gln-Phe	0.293	1.310	0.002	down
Val Ile Val	0.611	1.100	0.003	down
Met Trp	0.364	1.143	0.005	down
Cucurbitacin C	0.469	1.986	0.009	down
Phe Phe	0.651	1.084	0.001	down
P-Hydroxyubenimex	0.602	1.590	0.013	down
Arg-Thr-Lys-Arg	0.392	1.284	0.048	down
Tauro-B-Muricholic Acid	0.415	6.547	0.035	down
Oleanane -4h, + 2o	0.540	1.206	0.014	down
Soyasapogenol B 3-O-[A-L-Rhamnosyl-(1->4)-B-D-Galactosyl-(1->4)-B-D-Glucuronide]	0.521	2.745	0.040	down
Soyasaponin Ii	0.434	1.299	0.021	down
Soyasaponin V	0.417	1.363	0.009	down
Cincassiol B	0.415	1.362	0.022	down
Pe 22:2(13z,16z)/15:0	0.379	1.839	0.015	down

Table S2. Detailed information of differential metabolites ($|Log_2FC| > 1.5$, $P < 0.05$, and VIP > 1) between GG-PPC and Control groups.

Metabolite	FC	VIP	P value	Sig
N-Acetyl-Mannosamine	2.230	1.256	0.006	up
[(12-Methyltetraphen-7-Yl)Methoxy]Sulfonic Acid	5.979	1.203	0.001	up
4-(7-Methoxy-2-Oxochromen-6-Yl)Butan-2-Yl Hydrogen Sulate	8.957	1.475	0.000	up
5'-Carboxy-Gamma-Chromanol	2.237	1.277	0.002	up
Spirotryprostatin K	3.180	1.169	0.003	up
Lettucenin A	18.570	5.849	0.031	up
Prangenidin	27.967	1.714	0.037	up
5-Hydroxy-7-Methoxy-3-(3-Methoxyphenyl)-8-Methylchromen-4-One	2.043	7.114	0.029	up
Suc-Ala-Ala-Pro-Phe-P-Na	3.024	3.609	0.035	up
2-Butyrothienone	3.876	1.611	0.022	up
Pa(Pgf1alpha/20:3(8z,11z,14z))	23.956	1.230	0.000	up
Absintholide	4.752	3.845	0.002	up
7-O-2E-butenoyl macrolactin A	3.199	4.771	0.000	up
24,25-Epoxywithanolide D	6.489	3.904	0.006	up
Digoxigenin	5.315	1.104	0.007	up
[12]-Shogaol	2.294	1.985	0.006	up
Spiculisporic Acid	10.735	1.106	0.005	up
Sodium Deoxycholic Acid	2.110	2.064	0.034	up
Lpe 18:1	3.291	1.140	0.005	up
PG(16:0/0:0)[U]	2.618	8.201	0.048	up
Cpa(18:2(9z,12z)/0:0)	2.545	1.128	0.007	up
4-Ketoniridazole	15.801	3.464	0.004	up
Glucosamine 1-Phosphate	8.974	1.871	0.007	up
3-(2,4,5-trihydroxy-3-methoxyphenyl)propanoic acid	43.476	1.003	0.017	up
Mha Acid	1.557	1.692	0.024	up
7h-Purin-6-Amine	8.965	2.500	0.042	up
Galbeta1,3glcnac	3.983	2.800	0.013	up
Nicotine Acid Amide	4.460	1.113	0.002	up
Epicillin	3.793	3.336	0.039	up
Adenosine	2.832	6.220	0.002	up
Fenclonine	1.686	1.569	0.002	up
Dfdcmp	9.892	1.804	0.000	up
1,5-Isoquinolinediol	2.204	2.026	0.005	up

2-Amino-2-Deoxyisochorismate	2.119	3.490	0.007	up
(-)-Riboflavin	3.441	1.350	0.002	up
Trachelanthine	18.046	1.026	0.021	up
Ilicic Acid	2.840	1.355	0.009	up
Pd131628-0002b	2.174	1.009	0.005	up
3,4,5-trihydroxy-6-[4-(4-methyl-3-oxopent-1-en-1-yl)phenoxy]oxane-2-carboxylic acid	5.329	1.204	0.002	up
Blue Acid	6.839	1.004	0.000	up
N-Eicosapentaenoyl Tyrosine	3.036	1.921	0.003	up
D-Urobilinogen	48.157	1.250	0.008	up
1-B-D-Arabinosyl-5-Fluorouracil	4.144	1.319	0.014	up
N-Docosahexaenoyl Valine	3.386	2.101	0.001	up
Oligvon	6.545	1.236	0.008	up
Terragine B	12.521	1.606	0.000	up
Sterebin Q2	4.548	1.972	0.000	up
Hispidin	2.167	1.447	0.023	up
O-Phytodienoic Acid	1.734	1.590	0.004	up
Benzoic Acid,	1.974	2.200	0.000	up
Q-Y	16.398	1.812	0.050	up
Monoketocholic Acid	5.339	1.301	0.000	up
Cholic Acid	6.626	2.658	0.004	up
3'-C-Ethynylcytidine	14.070	1.155	0.013	up
Fe(II)-nicotianamine	24.379	1.857	0.012	up
Dodecylglycerol	4.937	1.054	0.029	up
Tetrahydroindol 5	54.806	1.034	0.020	up
Annomuricatin A	3.331	3.084	0.001	up
Meproscillarin	4.879	5.518	0.005	up
Ics 205-930	3.188	1.234	0.001	up
Pregnenediol	3.540	1.904	0.001	up
Oocyan	5.174	1.410	0.002	up
Incromega Dha 700esr	3.397	2.820	0.006	up
Geranicardic Acid	3.487	1.142	0.012	up
3-[1-(3-Cyclohexyl-3-Hydroxypropyl)-3,4-Dimethylpiperidin-4-Yl]Phenol	4.362	1.249	0.014	up
Pe(14:0/0:0)	2.262	7.097	0.019	up
Pc(14:0/0:0)	3.009	3.194	0.035	up
Ps(20:4/16:0)	4.801	2.120	0.005	up
Nae 15:0	2.525	2.577	0.011	up
Pc(18:1(12z)-O(9s,10r)/Dime(9,3))	5.418	2.580	0.001	up
13-Hydroxystearic Acid	1.976	6.168	0.026	up
Pe(22:6(4z,7z,11e,13z,15e,19z)-2oh(10s,17)/16:0)	13.952	1.482	0.000	up
Nonadecane-1,2,4-Triol	1.730	1.185	0.042	up
Lysope(16:1(9z)/0:0)	4.415	3.854	0.041	up

(13z,16z)-3-Hydroxydocosa-13,16-Dienoylcarnitine	11.705	1.023	0.022	up
Lysope(15:0/0:0)	2.787	14.544	0.014	up
Pa(20:0/0:0)	1.775	1.096	0.038	up
LysoPC(20:3(8Z,11Z,14Z))	4.820	1.954	0.030	up
Pe(16:0/0:0)	2.631	10.465	0.004	up
C1-13 Thermocryptoxanthin-13	3.213	2.075	0.008	up
N,N-Dimethylsphingosine	2.026	1.723	0.006	up
Oleoyl-L-Carnitine	3.494	4.449	0.006	up
Pe(18:1(9z)/0:0)	3.023	5.591	0.015	up
1-Hexadecyl-Glycero-3-Phosphate	2.430	8.442	0.001	up
Pe(17:0/0:0)	4.019	4.108	0.032	up
Aglepristone	3.979	9.026	0.000	up
Car 18:0	4.389	2.784	0.000	up
Gpc(17:0)	2.571	3.521	0.002	up
LPA(i-19:0/0:0)	4.343	1.964	0.011	up
Fty720 (S)-Phosphate	2.465	2.950	0.019	up
Lyso-PC(18:0/0:0)	2.671	12.434	0.002	up
N-Stearoyl Gaba	1.618	1.052	0.032	up
Iclaprim	2.157	1.035	0.039	up
Na-His 16:0	4.217	4.691	0.000	up
Cetyl Recinoleate	2.471	2.818	0.007	up
(Ch3)2chch2ch=Ch2	1.829	1.010	0.000	up
3beta-Hydroxy-4beta-Methyl-5alpha-Cholest-7-Ene-4alpha-Carboxylate	4.961	2.198	0.017	up
Butylamine	3.850	1.461	0.000	up
6)-Glucoside]	7.775	3.143	0.000	up
((+-))-(Z)-2-(5-Tetradecenyl)cyclobutanone	1.821	10.543	0.008	up
[6-(5,7-Dihydroxy-4-Oxo-2-Phenyl-4h-Chromen-8-Yl)-4,5-Dihydroxyoxan-3-Yl]Oxidanesulfonic Acid	0.217	1.601	0.000	down
Ribavirin Monophosphate	0.067	1.132	0.002	down
N-acetyl-S-(2-succino)-L-cysteine	0.048	1.493	0.006	down
Hexannic Acid	0.337	3.452	0.040	down
Leu-Gly-Gly	0.045	1.018	0.000	down
N-(2-Carbamoylethyl)Valine	0.161	1.145	0.001	down
Ala Ile	0.104	1.254	0.000	down
Tyrosyl-Glycine	0.038	1.182	0.000	down
5-Methoxycarbonyl-2-Thiophenecarboxylic Acid	0.482	1.006	0.033	down
L-Valic Acid	0.074	4.313	0.000	down
Resorcylic Acid	0.469	1.208	0.039	down
5-Amino-3-Oxohexanoate	0.305	1.386	0.018	down
Gamma-Glutamylleucine	0.107	1.538	0.002	down
Fema No. 3941	0.210	1.204	0.000	down

17-Phenyl-18,19,20-Trinor-Prostaglandin D2	0.021	1.595	0.003	down
Val Leu Ser Pro Ala	0.035	1.141	0.048	down
(4z,7z,10z)-12-[(1s,2r,3r)-3-Hydroxy-2-[(1e,3s)-3-Hydroxypent-1-En-1-Yl]-5-Oxocyclopentyl]Dodeca-4,7,10-Trienoylcarnitine	0.014	1.371	0.000	down
PS(12:0(3-OH)/12:0(3-OH))	0.000	1.000	0.001	down
5-Hydroxybuspirone	0.016	2.007	0.015	down
Cichorioside N	0.030	1.695	0.004	down
(S)-(alpha)-2-Hydroxyisocaproic acid	0.034	7.623	0.000	down
6-O-Caffeoylarbutin	0.322	2.651	0.001	down
N-Palmitoyl Aspartic acid	0.078	1.087	0.014	down
[3-(7-Hydroxy-4-Oxochromen-2-Yl)Phenyl] Hydrogen Sulate	0.014	4.095	0.047	down
[3-(5,7-Dihydroxy-4-Oxo-4h-Chromen-2-Yl)Phenyl]Oxidanesulfonic Acid	0.017	5.647	0.046	down
Sulfolithocholylglycine	0.149	1.680	0.015	down
Asperterpene H	0.264	1.064	0.018	down
Daidzein	0.142	3.141	0.025	down
Emodinol	0.341	1.260	0.012	down
Cytochalasin Opho	0.087	3.819	0.003	down
Dihydroactinidiolide	0.245	1.210	0.000	down
6-Gingesulfonic Acid	0.070	1.394	0.000	down
(R)-Equol	0.520	1.092	0.015	down
PZXDYQVSAFZTBI-UHFFFAOYSA-N	0.030	1.860	0.017	down
3-(4-Hydroxyphenyl)-3,5,6,8-Tetrahydro-2h-Chromene-4,7-Dione	0.342	2.546	0.043	down
(2s,3s,5s,8r,9s,10s,13s,14s,16s,17r)-17-Acetyloxy-10,13-Dimethyl-2-Morpholin-4-Yl-16-(1-Prop-2-Enylpyrrolidin-1-Ium-1-Yl)-2,3,4,5,6,7,8,9,11,12,14,15,16,17-Tetradecahydro-1h-Cyclopenta[A]Phenanthren-3-Olate	0.070	1.143	0.006	down
((+-))12,13-DiHOME	0.596	1.394	0.006	down
((+-))13-HpODE	0.340	2.939	0.045	down
(11s,12s,13s)-Epoxy-Hydroxyoctadeca-Cis-9-Cis-15-Dien-1-Oic Acid	0.381	1.402	0.012	down
Alpha-Dimorphecolic Acid	0.422	1.700	0.001	down
5,6-Dehydro Aa	0.438	1.277	0.005	down
Cervonic Acid	0.439	1.320	0.002	down
4h-1-Benzopyran-4-One, 5,7-Dihydroxy-2-(1-Hydroxy-4-Oxo-2,5-Cyclohexadien-1-Yl)-	0.401	1.361	0.039	down

[4-[2,3-Dioxo-3-(2,4,6-Trihydroxy-3-Methoxyphenyl)Propyl]-2-Hydroxy-6-Methoxyphenyl] Hydrogen Sulate	0.370	1.275	0.049	down
Zoledronic Acid	0.194	1.085	0.001	down
N-Acetylgalactosamine 4-Sulphate	0.399	2.558	0.020	down
N-Acetylhistamine	0.136	3.323	0.008	down
D-Arg	0.226	2.408	0.000	down
Glutaminylvaline	0.123	2.087	0.000	down
Phe Thr Gly	0.137	1.266	0.042	down
Val Val	0.301	1.150	0.000	down
Alanylleucine (isomer of 675)	0.163	2.994	0.000	down
Ser Leu	0.223	2.442	0.000	down
Trp-Thr	0.173	1.167	0.007	down
Homopantothenic Acid	0.243	1.953	0.000	down
Asn Leu	0.097	2.662	0.000	down
Leucylglutamic Acid	0.153	2.606	0.000	down
Ile Asp Thr	0.234	1.032	0.018	down
Aconine	0.005	2.066	0.000	down
Isoleucylglutamic Acid	0.168	1.652	0.009	down
Gly Phe	0.272	1.484	0.007	down
Delta-Hydroxy-Lys-Nle	0.117	1.187	0.010	down
Chrysopiperazine C	0.029	1.185	0.001	down
Daumone	0.040	2.341	0.001	down
3'-Hydroxyropivacaine	0.052	3.739	0.002	down
Ala Glu Leu	0.141	1.590	0.000	down
2-Hydroxyacorenone	0.082	1.074	0.018	down
Isofloxythepin	0.050	2.606	0.005	down
6-O-Oleuropeoylsucrose	0.187	1.138	0.011	down
5-Hpq	0.025	2.178	0.000	down
S-(3-Methylbutanoyl)-Dihydrolipoamide	0.067	1.019	0.008	down
Brevianamide A	0.129	1.817	0.003	down
F-V	0.242	2.053	0.000	down
3-(5-Methylfurfurylidene)-1-Pyrroline	0.087	2.357	0.010	down
Miotoxin A	0.029	1.075	0.000	down
3-(4-cyclohexylpiperazine-1-carbonyl)-2-methyl-5-(2-methylpropyl)thieno[3,2-c]pyridin-4-one	0.064	1.912	0.000	down
Val Ser Phe	0.133	1.107	0.001	down
Lyciumoside I	0.000	1.008	0.000	down
Isoleucylleucine	0.130	3.276	0.000	down
Arg-Arg-Gln-Phe	0.018	1.032	0.000	down
Val Ile Val	0.184	1.059	0.000	down
Streptothricin F	0.056	1.165	0.002	down
Ethyl ((3,4,5-Trihydroxy-6-((4-(4-Isopropoxybenzyl)-1-Isopropyl-5-Methyl-1h-	0.046	1.272	0.000	down

Pyrazol-3-Yl)Oxy)Tetrahydro-2h-Pyran-2-Yl)Methyl) Carbonate				
Gln Ile Ile	0.182	2.452	0.000	down
Cyclosquamosin B	0.033	1.185	0.002	down
Leu Ala Ile	0.181	1.736	0.000	down
[4-(2-ethyl-2,3-dihydroimidazo[1,2-c]pyrimidin-7-yl)piperazin-1-yl]-(1-methylindazol-3-yl)methanone	0.033	1.278	0.001	down
11,13-Dihydrotaraxinic Acid Glucosyl Ester	0.132	4.296	0.009	down
Cucurbitacin C	0.009	1.932	0.000	down
Rhizoxin M1	0.007	1.361	0.000	down
Phe Phe	0.135	1.138	0.000	down
Val Ile Leu	0.284	1.325	0.000	down
Asp Ile Leu	0.237	1.203	0.008	down
P-Hydroxyubenimex	0.081	1.745	0.000	down
Ac-Ser-Asp-Lys-Pro-Oh	0.008	1.522	0.003	down
Ala Leu Phe	0.182	1.614	0.003	down
Citroside A	0.067	1.761	0.000	down
Arg-Thr-Lys-Arg	0.000	1.226	0.001	down
Val Leu Ala Leu Leu	0.050	1.412	0.000	down
Sterhirsutin I	0.014	1.162	0.000	down
Neocasomorphin	0.018	1.715	0.000	down
Metolachlor Morpholinone	0.379	1.047	0.021	down
3-[(1e)-Buta-1,3-Dienyl]-5-Hydroxyisochromen-1-One	0.231	4.458	0.029	down
Tauro-B-Muricholic Acid	0.273	4.752	0.017	down
Oleanane -4h, + 2o	0.126	1.125	0.002	down
Soyasapogenol B 3-O-[A-L-Rhamnosyl-(1->4)-B-D-Galactosyl-(1->4)-B-D-Glucuronide]	0.113	2.670	0.003	down
Soyasaponin Ii	0.109	1.119	0.003	down
Goyaglycoside H	0.096	2.132	0.006	down
Soyasaponin V	0.030	1.178	0.000	down

Table S3. Matrix for correlation analysis.

	g__Bacteroides	g__Alistipes	g__Alloprevotella	g__Lactobacillus	g__Lachnospiraceae	g__Roseburia	g__Helicobacter	g__Akkermansia	g__Enterorhabdus	g__Erysipelatoclostridium	g__Muribaculaceae	(S)-(alpha)-2-Hydroxyisocaproic acid	Aglepristone	1-Hexadecyl-Glycero-3-Phosphate	Tauro-B-Muricholic Acid	Adenosine	L-Valic Acid	Cytochalasin Opho	8-Hydroxy-7(11)-Eremophilen-12,8-Olide	Pc(14:0/0:0)	Lysope(15:0/0:0)	Lyso-PC(18:0/0:0)	((+-))-(Z)-2-(5-Tetradecenyl)cyclobutanone	Pe(16:0/0:0)	PG(16:0/0:0)[U]	5-Hydroxy-7-Methoxy-3-(3-Methoxyphenyl)-8-Methylchromen-4-One	Pe(14:0/0:0)
Total carbohydrates content	0.06667	-0.46667	-0.73333	-0.2	-0.6	-0.6	-0.33333	0.33333	0.6	0.86667	0.73333	-0.46667	-0.86667	-0.6	-0.2	1	-0.44721	-0.89443	-0.44721	-0.89443	0.89443	0.44721	0.44721	0.74536	0.89443	0.44721	0.89443
Protein content	-0.2582	0.7746	0.7746	0.7746	0.7746	0.7746	0.7746	-0.7746	-0.7746	-0.7746	-0.7746	0.7746	0.7746	0.7746	0.7746	-0.7746	0.86603	0.86603	0.86603	0.86603	-0.86603	-0.86603	-0.86603	-0.86603	-0.86603	-0.86603	-0.86603
Uronic acid content	0.2582	-0.7746	-0.7746	-0.7746	-0.7746	-0.7746	-0.7746	0.7746	0.7746	0.7746	0.7746	-0.7746	-0.7746	-0.7746	-0.7746	0.7746	-0.86603	-0.86603	-0.86603	-0.86603	0.86603	0.86603	0.86603	0.86603	0.86603	0.86603	0.86603
Fuc	-0.46667	0.86667	0.33333	0.6	0.2	0.73333	0.73333	-0.73333	-0.2	-0.73333	-0.86667	0.86667	0.46667	1	0.6	-0.6	0.44721	0.89443	0.44721	0.89443	-0.44721	-0.89443	-0.89443	-0.59628	-0.44721	-0.89443	-0.44721
GalN	0.33333	-0.73333	-0.46667	-0.46667	-0.33333	-0.86667	-0.6	0.6	0.33333	0.6	1	-0.73333	-0.6	-0.86667	-0.46667	0.73333	-0.44721	-0.89443	-0.44721	-0.89443	0.59628	0.74536	0.74536	0.74536	0.59628	0.74536	0.59628
Rha	0.06667	0.33333	0.86667	0.6	1	0.46667	0.46667	-0.46667	-1	-0.46667	-0.33333	0.33333	0.73333	0.2	0.6	-0.6	0.89443	0.44721	0.89443	0.44721	-0.89443	-0.44721	-0.44721	-0.74536	-0.89443	-0.44721	-0.89443
Ara	-0.06667	-0.33333	-0.86667	-0.6	-1	-0.46667	-0.46667	0.46667	1	0.46667	0.33333	-0.33333	-0.73333	-0.2	-0.6	0.6	-0.89443	-0.44721	-0.89443	-0.44721	0.89443	0.44721	0.44721	0.74536	0.89443	0.44721	0.89443
GlcN	-0.33333	0.73333	0.46667	1	0.6	0.6	0.86667	-0.86667	-0.6	-0.33333	-0.46667	0.73333	0.33333	0.6	1	-0.2	0.89443	0.44721	0.89443	0.44721	-0.44721	-0.89443	-0.89443	-0.59628	-0.44721	-0.89443	-0.44721
Gal	-0.46667	0.86667	0.33333	0.6	0.2	0.73333	0.73333	-0.73333	-0.2	-0.73333	-0.86667	0.86667	0.46667	1	0.6	-0.6	0.44721	0.89443	0.44721	0.89443	-0.44721	-0.89443	-0.89443	-0.59628	-0.44721	-0.89443	-0.44721

Glc	0.2	0.2	1	0.46667	0.86667	0.6	0.6	-0.6	-0.86667	-0.6	-0.46667	0.2	0.6	0.33333	0.46667	-0.73333	0.74536	0.59628	0.74536	0.59628	-0.89443	-0.44721	-0.44721	-0.74536	-0.89443	-0.44721	-0.89443
Xyl	-0.06667	0.46667	0.73333	0.2	0.6	0.6	0.33333	-0.33333	-0.6	-0.86667	-0.73333	0.46667	0.86667	0.6	0.2	-1	0.44721	0.89443	0.44721	0.89443	-0.89443	-0.44721	-0.44721	-0.74536	-0.89443	-0.44721	-0.89443
Man	-0.46667	0.86667	0.33333	0.6	0.2	0.73333	0.73333	-0.73333	-0.2	-0.73333	-0.86667	0.86667	0.46667	1	0.6	-0.6	0.44721	0.89443	0.44721	0.89443	-0.44721	-0.89443	-0.89443	-0.59628	-0.44721	-0.89443	-0.44721
Rib	0.44721	-0.89443	-0.44721	-0.89443	-0.44721	-0.74536	-0.89443	0.89443	0.44721	0.59628	0.74536	-0.89443	-0.44721	-0.89443	-0.89443	0.44721	-0.75	-0.75	-0.75	-0.75	0.5	1	1	0.66667	0.5	1	0.5
GalA	0	-0.44721	-0.89443	-0.44721	-0.89443	-0.59628	-0.44721	0.44721	0.89443	0.74536	0.59628	-0.44721	-0.89443	-0.44721	-0.44721	0.89443	-0.75	-0.75	-0.75	-0.75	1	0.5	0.5	0.83333	1	0.5	1
GlcA	-0.06667	-0.33333	-0.86667	-0.6	-0.73333	-0.46667	-0.73333	0.73333	0.73333	0.73333	0.33333	-0.33333	-0.46667	-0.46667	-0.6	0.6	-0.74536	-0.59628	-0.74536	-0.59628	0.74536	0.59628	0.59628	0.59628	0.74536	0.59628	0.74536
Mw	-0.06667	-0.33333	-0.86667	-0.6	-1	-0.46667	-0.46667	0.46667	1	0.46667	0.33333	-0.33333	-0.73333	-0.2	-0.6	0.6	-0.89443	-0.44721	-0.89443	-0.44721	0.89443	0.44721	0.44721	0.74536	0.89443	0.44721	0.89443
Mn	0.6	-1	-0.2	-0.73333	-0.33333	-0.6	-0.6	0.6	0.33333	0.6	0.73333	-1	-0.6	-0.86667	-0.73333	0.46667	-0.59628	-0.74536	-0.59628	-0.74536	0.44721	0.89443	0.89443	0.59628	0.44721	0.89443	0.44721

Note: Red markings indicate significant differences.
